# Procalcitonin as a predictor of length of stay in patients with odontogenic infections

**DOI:** 10.1007/s10006-026-01617-6

**Published:** 2026-08-07

**Authors:** Felix Thol, Felix Benjamin Warwas, Nikolai Spuck, Anne Klausing, Tim Klünter, Paul Stoll, Franz-Josef Kramer, Nils Heim

**Affiliations:** 1 Clinic and Polyclinic for Oral, Maxillofacial, and Plastic Facial Surgery, University Hospital Bonn, Venusberg-Campus 1, Building 11, 2. OG, Bonn, D-53127 Germany; 2 Core Facility Biostatistics, Medical Faculty Mannheim, Central Institute of Mental Health, Heidelberg University, Mannheim, J5, 68159 Germany; 3 Institute of Medical Biometry, Informatics, and Epidemiology, University Hospital Bonn, Baunscheidstraße 17, Bonn, 53127 Germany

**Keywords:** Odontogenic abscesses, Procalcitonin, PCT, Length of stay, LOS

## Abstract

**Objectives:**

Our study aimed to evaluate procalcitonin (PCT) as a prognostic marker in advanced odontogenic infections. In addition, we sought to identify further prognostic markers that could be used to predict the length of hospital stay (LOS).

**Materials and methods:**

We retrospectively analysed 61 patients who were hospitalised between January and August 2020 for surgical abscess drainage in the Department of Oral and Maxillofacial Surgery at the University Hospital of Bonn. Univariable logistic discrete hazard models were used to assess the association between baseline levels as well as temporal changes in laboratory parameters (PCT, C-reactive protein (CRP), and leukocytes) and LOS. In addition, a tree-based approach for modelling discrete time-to-event data was used to identify risk groups of patients with a prolonged LOS.

**Results:**

Median LOS were 5, 7, and 9 days for different PCT concentrations < 0.1 µg/l, 0.1–0.5 µg/l, and > 0.5 µg/l, respectively (*p* < 0.001). The data-driven tree identified three subgroups with median LOS of 4, 6, and 10 days based on preoperative CRP and PCT levels. Patients with CRP > 153 mg/l had a median LOS of 10 days, while those with CRP ≤ 153 mg/l had median LOS of 6 or 4 days depending on whether PCT was > 0.03 µg/l or ≤ 0.03 µg/l.

**Conclusions:**

PCT may serve as a useful prognostic marker for estimating LOS, even in severe odontogenic infections. Exploratory decision tree analysis demonstrated that the combined assessment of PCT and CRP levels could help stratify patients into distinct LOS subgroups, which may facilitate clinical decision-making and more accurate estimation of treatment outcomes.

**Supplementary Information:**

The online version contains supplementary material available at 10.1007/s10006-026-01617-6.

## Introduction

Odontogenic infections originate in the teeth or periodontium [1]. If the inflammatory reaction progresses from apical periodontitis, subperiosteal bone areas are initially affected, followed by various soft tissue regions [2]. As the infection spreads, life-threatening complications such as necrotising fasciitis, mediastinitis or sepsis are not uncommonly seen [3, 4]. Due to the incidence of these infections, in addition to the dangers to the individual, high economic costs are often incurred, mainly due to surgical interventions, the length of hospital stay (LOS) or intensive care treatments [5–8].

Predicting treatment outcomes and managing therapy is challenging for treating physicians, especially in cases of extensive infections [9, 10]. Due to a lack of improvement or worsening symptoms, it is not uncommon that revision surgeries or transfers to the intensive care unit are necessary, which significantly prolongs the LOS [11].

To support decision-making in everyday clinical practice, prognostic markers such as procalcitonin (PCT) have become established in various specialist areas and are routinely used for risk stratification in patients with severe respiratory tract infections [12] or sepsis [13]. Despite the potentially life-threatening nature of odontogenic infections, evidence on the clinical value of PCT for their assessment remains scarce [14–16].

Improved prognostic assessment could bring various advantages, such as earlier identification of risk groups with accelerated referral to further diagnostic and therapeutic measures. At the same time, costs would be minimised, as hospital stays could be better estimated and patients could be discharged earlier.

This study therefore aimed to investigate the use of PCT as a predictive marker in patients with odontogenic infections. In addition, further prognostic parameters should be identified in order to improve patient outcomes and reduce economic costs in the future.

## Methods

### Patients & methods

We conducted a retrospective study involving 61 patients who were treated for odontogenic abscesses at the Department of Oral and Maxillofacial Plastic Surgery at the University Hospital Bonn between January 2020 and August 2020. Inclusion criteria comprised the presence of an abscess with a tendency for further spread requiring inpatient treatment with intravenous antibiotic therapy. In addition, surgical management by either intraoral or extraoral drainage of the lesion had to be performed. Furthermore, at least one preoperative blood sample was required for the assessment of the investigated laboratory parameters, including PCT, C-reactive protein (CRP), and leukocyte count.

Odontogenic abscesses were diagnosed by clinical examination. Preoperative CT imaging was reserved for cases with diagnostic uncertainty or suspected extensive infection spread.

In order to evaluate the laboratory parameters in terms of their usefulness as predictive markers and to stratify the patient population, various parameters were collected from the clinical information systems. In addition to LOS, other factors such as age at the operation, discharge of pus during abscess incision, and the need for revision were assessed.

This study was conducted in accordance with the Declaration of Helsinki of 1964 and approved by the Ethics Committee of the University of Bonn (reference 2026-122-BO). As retrospective studies have only been required to undergo review by the Ethics Committee since the amendment to Sect.  15(1) of the North Rhine Medical Regulations in July 2025, the study was submitted once data collection had been completed. The relevant documents are available in the supplementary material.

### Statistical analysis

Statistical analysis was performed using R version 4.3.1 (R Core Team, 2021, Vienna, Austria) and comprised two parts. The first part focused on investigating the association between inflammatory parameters and incidence of pus during abscess incision. Specifically, the differences in CRP (mg/l), leukocytes (g/l) and PCT (µg/l) between patients with and without incidence of pus were compared based on a Wilcoxon-Mann-Whitney test. Additionally, the association between PCT category (normal (0): <0.1 µg/l; moderately elevated (1): 0.1–0.5 µg/l; elevated (2): >0.5 µg/l) was evaluated based on Fisher’s exact test. Prior to analysis, one PCT value (> 60 µg/l) was excluded because of concerns regarding data validity and clinical plausibility. This decision was based on its marked deviation from all other measured PCT values (< 2 µg/l) as well as the absence of clinical complications, systemic infection, or septic disease in the respective patient. This exclusion was also applied to the analyses of LOS described below.

In the second part of the analysis, LOS in hospital was considered. The objective was to investigate whether LOS was affected by the inflammatory parameters. For this, a time-to-event model was applied, where the event of interest was discharge from hospital and time was measured in days. More specifically, the data were analysed using univariable logistic discrete hazard models, which are common tools for the analysis of discrete time-to-event data. In these models, the conditional probability

$$\:\lambda\:\left(t\right)=P\left(T=t\right|\:T>t,\:x),$$

where *T* is the time the event of interest occurs and *x* is a variable possibly affecting the probability for an event, is analyzed. That is, in the model the conditional probability that the event of interest occurs at time *t*, given that it has not occurred up to that time point *t*, is estimated. Here, the effect of time since admission was modelled in a flexible way via a smooth function and a linear effect of each inflammatory parameter was included in each univariable model. In particular, a separate model was applied for CRP, leukocytes, PCT and PCT category as well as for change from the preoperative to the postoperative blood sample value in CRP, leukocyte and PCT, respectively. An additional objective of the analysis was to identify risk groups of patients with a prolonged LOS. In order to do so, a tree-based approach for modelling discrete time-to-event data by Spuck et al. (2023) was used [17]. This method also applies the logistic discrete hazard model framework but allows to detect subgroups of patients with different probabilities for the occurrence of an event in data-driven way. Analogously to the univariable logistic discrete hazard models, the effects of time since admission were included as a flexible smooth function. CRP, leukocytes and PCT were included as possible splitting variables in the tree building. The change in the inflammatory parameters was not included as the postoperative values were not available for all patients resulting in a larger amount of missing values. The optimal splitting rules based on these variables were selected by minimizing the deviance. The size of the tree determining the risk groups was optimized using the Bayesian information criterion [18, 19]. To avoid overfitting, the maximal number of splits considered was set to 5 and the minimal number of observations in each node was bounded downward by 15% of the size of the fitting data. For evaluation of the stability of the tree-based model, it was refitted to 500 bootstrap samples and selection rates for the candidate splitting variables averaged across the bootstrapped models were calculated. For more details on discrete hazard models, see Tutz and Schmid (2016) and Berger and Schmid (2018) [20, 21].

## Results

### Baseline characteristics

The sex distribution of the cohort was balanced. With regard to the soft tissue compartments affected, the perimandibular and submandibular compartments were most commonly affected. These accounted for approximately half of the cohort. Other abscess locations, such as pterygomandibular abscesses, were less common.

The dental focus of the abscesses was most frequently located in the area of the lower mandibular teeth. A focus in the area of the upper jaw and the anterior teeth occurred less frequently. In approximately one quarter of the cohort, no definite dental focus could be identified. A summary of the baseline characteristics and the dental foci can be found in Tables 1 and 2.

**Table 1:** Baseline characteristics and frequency of different abscess localisations. Continuous variables are expressed as medians with interquartile ranges in brackets, categorical variables as absolute and relative frequencies (%)

Baseline characteristics	Patients
Age at operation (years)	51.9 (29.8, 68.1)
Abscess localisation	
Perimandibular	17 (27.9%)
Submandibular	13 (21.3%)
Pterygomandibular	9 (14.8%)
Submental	6 (9.8%)
Paramandibular	6 (9.8%)
Cheek	4 (6.6%)
Canine fossa	2 (3.3%)
Massertericomandibular	2 (3.3%)
Other	2 (3.3%)

**Table 2:** Dental focus of the abscesses (FDI tooth designation). Molar teeth of the lower jaw were the most common cause. The category “not clearly defined” comprises cases with incomplete information or infections in which multiple teeth were considered a possible source of the abscess. Categorical variables are expressed as absolute and relative frequencies (%)

Teeth	Patients
18	1 (1.6%)
23	1 (1.6%)
31	1 (1.6%)
33	4 (6.6%)
34	2 (3.3%)
35	3 (4.9%)
36	5 (8.2%)
37	4 (6.6%)
38	4 (6.6%)
41	1 (1.6%)
43	1 (1.6%)
44	2 (3.3%)
45	1 (1.6%)
46	5 (8.2%)
47	5 (8.2%)
48	6 (9.8%)
Not clearly defined	15 (24.6%)

## Treatment outcome

All patients underwent preoperative blood sampling during their hospital admission. The median values for inflammatory parameters such as CRP, leukocytes and PCT were 85.00 mg/l, 13.68 g/l and 0.06 µg/l, respectively. With regard to PCT concentrations, approximately two-thirds of the cohort had normal concentrations < 0.1 µg/l, almost a quarter had moderately elevated concentrations of 0.1–0.5 µg/l and approximately 7% had elevated values above 0.5 µg/l. Follow-up blood samples were taken at a median of 2 days after the first preoperative blood sample. The CRP, leukocyte and PCT concentrations decreased by a median of 34.00 mg/l, 3.35 g/l and 0.02 µg/l, respectively. A summary of all therapy parameters can be found in Table 3.

**Table 3:** Treatment outcomes. Continuous variables are expressed as medians with interquartile ranges in brackets, categorical variables as absolute and relative frequencies (%); Asterisks indicate missing values; */ *n* = 60 ** /*n* = 52; *** / *n* = 58

Variable	Summary statistic (*n* = 61)
CRP (mg/l)	85.00 (42.00, 144.00)
Leukocytes (g/l)	13.68 (10.90, 16.80)
Procalcitonin (µg/l) *	0.06 (0.03, 0.10)
Procalcitonin categorised *	
< 0.1 (µg/l)	42 (70.0%)
0.1–0.5 (µg/l)	14 (23.3%)
> 0.5 (µg/l)	4 (6.7%)
CRP change (mg/l) **	-34.00 (-80.00, 1.50)
Leukocytes change (g/l) **	-3.35 (-6.00, -1.07)
Procalcitonin change (µg/l) **	-0.02 (-0.04, 0.00)
Time between sample collection (days) **	2 (2.0, 3.0)
Pus drainage during abscess incision ***	40 (69.0%)
Surgical revision	6 (9.8%)
Length of stay (days)	6.0 (4.0, 7.0)

**Fig. 1:**
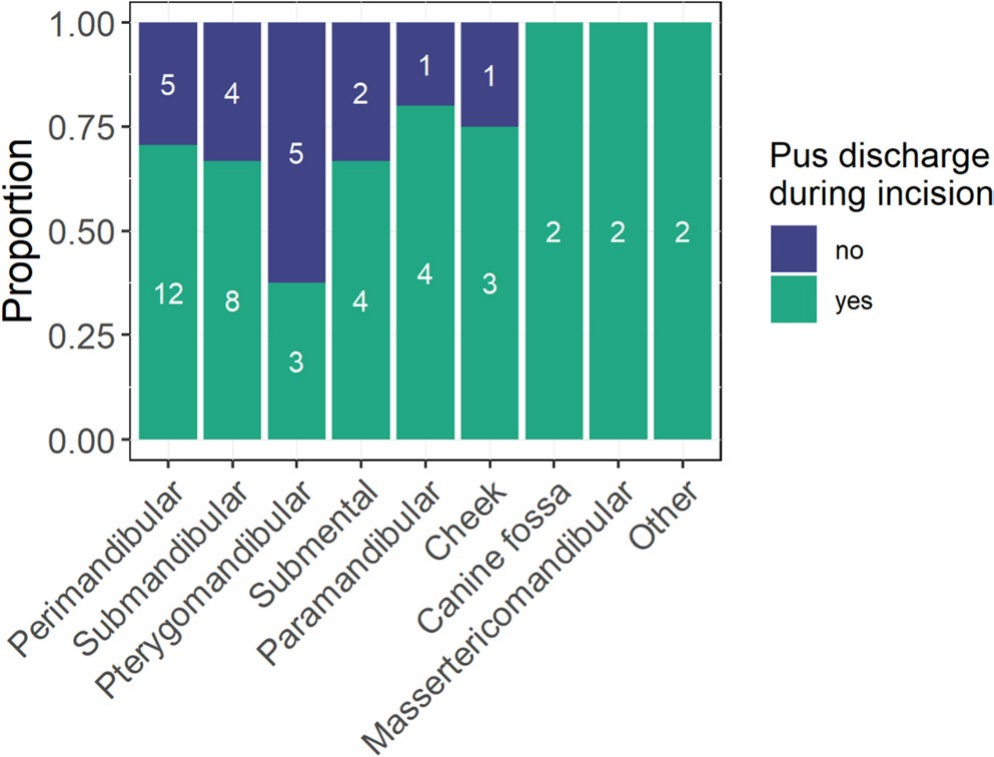
Incidence of pus discharge during incision for different abscess types. Pterygomandibular abscesses were associated with the highest failure rate

Following hospital admission, intraoral or extraoral abscess drainage was performed promptly. In the course of this, pus discharge was observed during abscess incision in almost two-thirds of the cohort. The frequency of pus presence during abscess incision depended on the affected soft tissue compartment. In this context, pterygomandibular abscesses were the least likely to drain pus (Fig. 1).

The diagnostic distinction between an infiltrate and an abscess could not be supported by blood values such as CRP (*p* = 0.444), leukocytes (*p* = 0.886), PCT (*p* = 0.252) and PCT category (*p* = 0.886). That is, no significant differences in the concentrations of inflammatory parameters between patients with and without incidence of pus during abscess incision were observed.

Revision surgery was necessary in approximately 10% of patients due to a lack of improvement after the first operation. No patient required monitoring in the intensive care unit.

The overall median LOS was 6 days. The presence of pus drainage during abscess incision did not significantly affect the conditional probability of discharge from hospital (*p* = 0.324).

**Fig. 2:**
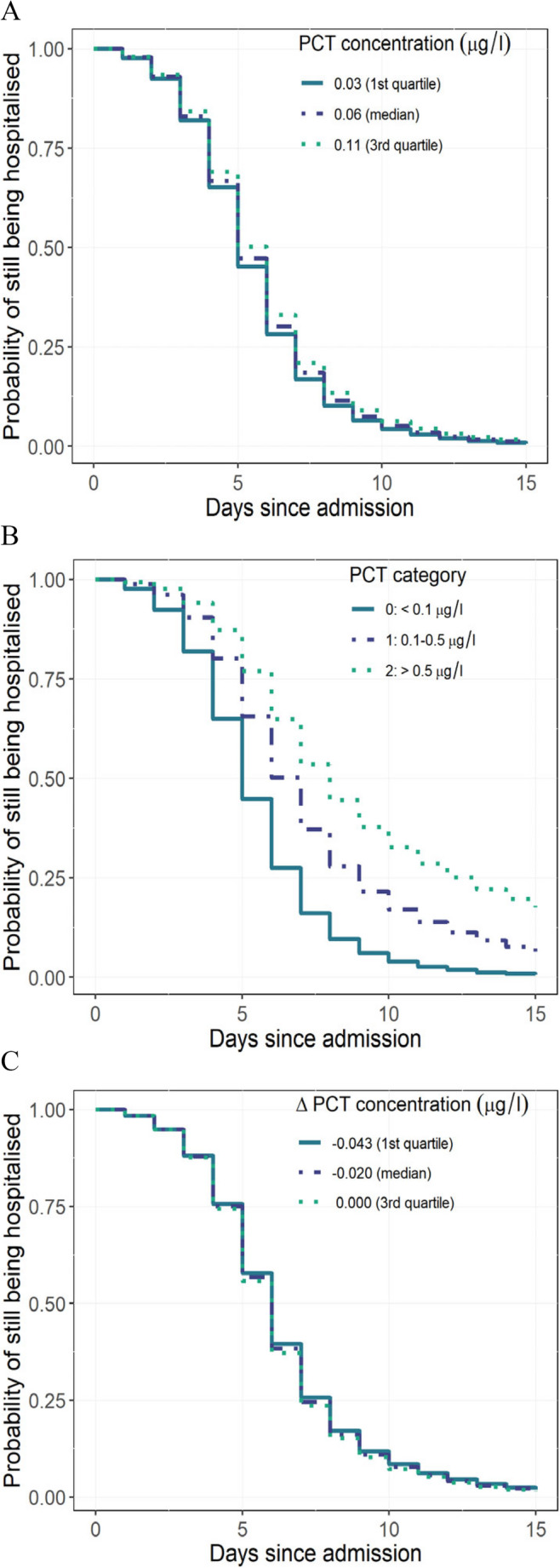
(**a**) LOS depending on different PCT concentrations. First quartile: 0.03 µg/l (blue), median: 0.06 µg/l (purple), third quartile: 0.11 µg/l (green). The median LOS was 5, 5 and 6 days and a significant association was found (*p*=0.024) (**b**) LOS according to baseline PCT levels. Category 0: <0.1 µg/l (blue), Category 1: 0.1–0.5 µg/l (purple), Category 2: >0.5 µg/l (green). Median LOS increased from 5 to 9 days and differed significantly between groups (*p*<0.001) (**c**) LOS according to changes in PCT levels based on sample quantiles. First quartile: −0.043 µg/l (blue), median: −0.020 µg/l (purple), third quartile: 0.000 µg/l (green). No significant association with LOS was observed (*p*=0.058)

A linear effect of PCT measured in µg/l on the probability of discharge was shown with median LOS of 5 days for the first PCT quartile (0.03 µg/l) and the median (0.06 µg/l) as well as a median LOS of 6 days for the third PCT quartile (0.11 µg/l; *p* = 0.024; Fig. 2a). Modelling the effect of PCT on the probability of discharge with a smooth nonlinear P-spline function also yielded a significant result (*p* = 0.001). LOS also varied across the PCT categories, with lengths of stay of 5 days (category 0), 7 days (category 1) and 9 days (category 2), representing a significant difference (*p* < 0.001; Fig. 2b). The change in PCT concentration over time showed no significant association with LOS (*p* = 0.058; Fig. 2c). 

**Fig. 3:**
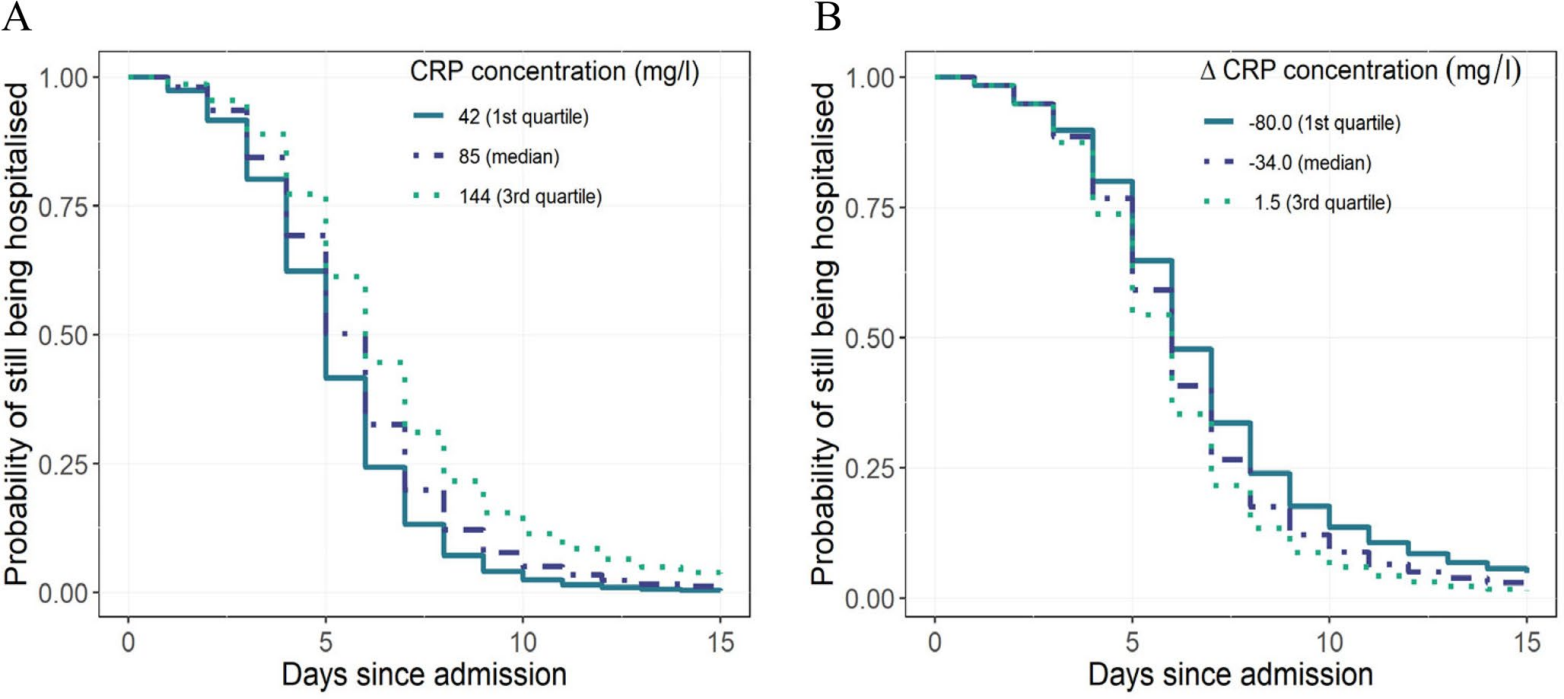
(**a**) LOS according to baseline CRP levels. First quartile: 42 mg/l (blue), median: 85 mg/l (purple), third quartile: 144 mg/l (green). The median LOS was 5, 6 and 6 days and a significant association was found (*p*=0.003) (**b**) LOS according to changes in CRP levels based on sample quantiles. First quartile: −80.0 mg/l (blue), median: −34.0 mg/l (purple), third quartile: 1.5 mg/l (green). No significant association with LOS was observed (*p*=0.055)

Preoperative CRP concentrations also had a significant influence on the probability of discharge, with median LOS of 5, 6 and 6 days for CRP values at the first quartile (42 mg/l), median (85 mg/l), and third quartile (144 mg/l), respectively (p=0.003; Fig. 3a). The change in CRP concentrations also had no significant association with LOS (*p*=0.055; Fig. 3b). 

**Fig. 4:**
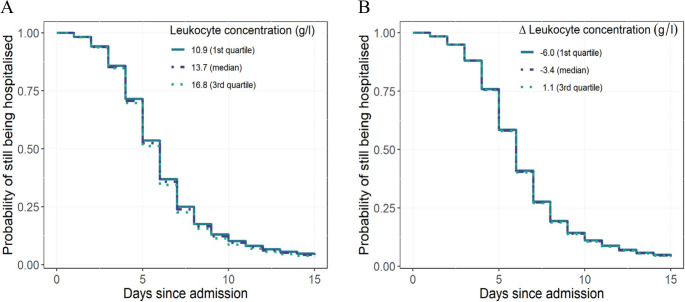
(**a**) LOS according to baseline leukocyte levels. First quartile: 10.9 g/l (blue), median: 13.7 g/l (purple), third quartile: 16.8 g/l (green). No significant association with LOS was observed (*p*=0.375) (**b**) LOS according to changes in leukocyte levels based on sample quantiles. First quartile: −6.0 g/l (blue), median: −3.4 g/l (purple), third quartile: 1.1 g/l (green). No significant association with LOS was observed (*p*=0.896)

Neither the initial leukocyte concentrations (p=0.375; Fig. 4a) nor the change from baseline to follow-up showed any association with the LOS (*p*=0.896; Fig. 4b). 

**Fig. 5:**
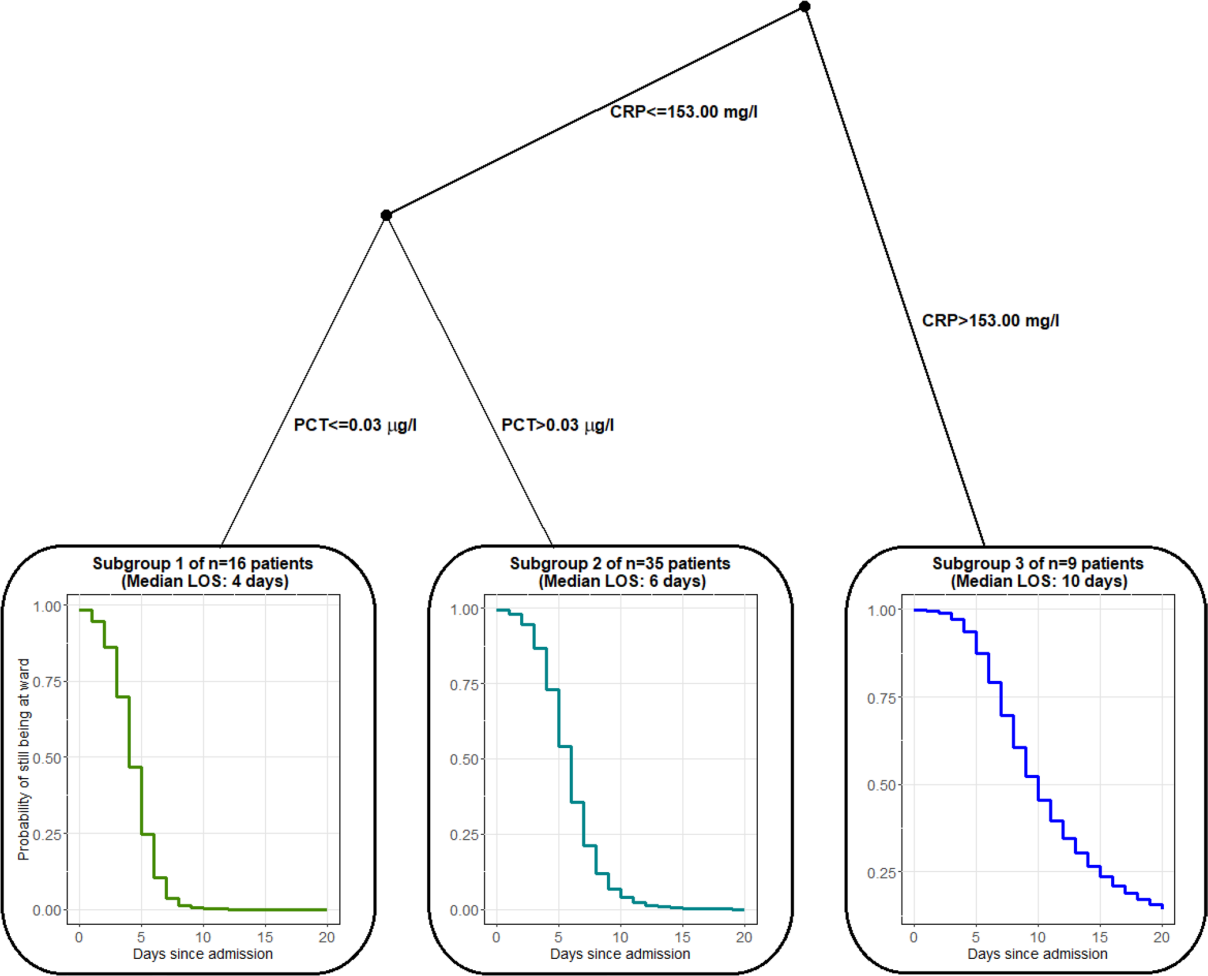
Exploratory decision tree model for LOS prediction based on CRP and PCT levels. Median LOS was 10 days for CRP >153 mg/l (blue), 6 days for CRP ≤153 mg/l and PCT >0.03 µg/l (light blue), and 4 days for CRP ≤153 mg/l and PCT ≤0.03 µg/l (green)

The data-driven tree building procedure identified three subgroups with median LOS of 4, 6 and 10 days based on the preoperative CRP and PCT concentration levels. If preoperative CRP concentrations were above 153 mg/l, the median LOS was 10 days. For CRP concentrations below 153 mg/l, PCT concentrations were selected for further differentiation. For values above 0.03 µg/l, the median LOS was 6 days, compared to 4 days for a subgroup with values below 0.03 µg/l (Fig. 5). PCT and CRP concentration were selected as a splitting variable in 83% and 54% of the bootstrap samples, respectively, whereas only 2% of the bootstrapped trees contained leukocytes as a splitting variable. These results indicate notable variability of the tree structure but support our findings from the univariable analyses. 

## Discussion

Odontogenic infections arise from deeply decayed teeth, cysts or after tooth extraction [22]. The underlying spectrum of pathogens originates from the oral microbiome [23, 24]. Depending on the various stages of infection, either practising dentists or oral and maxillofacial surgery clinics are involved in their treatment [6, 25]. Abscesses with a tendency to spread should be treated with surgical drainage and intravenous antibiotic therapy in an inpatient setting [26]. Precisely these abscesses often lead to complications during therapy, which makes it difficult for the treating physician to anticipate the outcome and manage treatment [27–29]. In order to better predict the progression of patients with infectious diseases, predictive markers such as PCT or interleukin 6 have already become established in various other specialties. PCT was first used for risk stratification of patients with sepsis and has now become firmly established in emergency and intensive care medicine [12, 13]. Currently, practitioners treating odontogenic infections cannot rely on such additional decision-making criteria, which can lead to delayed identification of high-risk groups and prolonged hospital stays for mildly affected patients. Our study therefore aimed to evaluate the value of PCT as a predictive marker in extensive odontogenic infections and to identify further prognostic factors in order to improve the evidence for treating physicians in the future.

The course of action regarding appropriate treatment is determined during the initial contact with the patient. Thus, the presence of an infiltrate can be addressed with oral antibiotics, while patients with an abscess should undergo surgical drainage. Furthermore, if the abscess is spreading, the patient should be referred for inpatient treatment [26]. Decisions for or against surgery are primarily based on clinical experience and supported by laboratory parameters such as leukocytes or CRP. Whether laboratory parameters improve the differential diagnosis between infiltrate and abscess remains questionable, as our study data showed no association between inflammatory parameters (CRP, leukocytes and PCT) and the presence of pus during abscess incision. The distinction between infiltrate and abscess, on the other hand, was influenced by the location of the inflammation. In cases of pterygomandibular abscesses, surgical drainage more frequently failed to yield pus despite a clinical diagnosis of an abscess. However, it remains to be seen whether this diagnostic differentiation has an impact on treatment outcomes or whether both patients with infiltrates and abscesses benefit from drainage with lavage. Thus, our analyses did not show any significant effect of the presence of pus during abscess incision on the LOS.

In order to predict the LOS and better assess the discharge eligibility of patients, previous studies have attempted to identify prognostic markers for patients with odontogenic abscesses [7, 10, 30, 31]. Among inflammatory markers, CRP in particular has repeatedly been identified as a key parameter, with both CRP and leukocyte concentration being associated with prolonged hospitalization and increased recurrence rates [14–16, 32–35] Furthermore, a limited number of studies have recently begun to investigate the potential utility of other inflammatory biomarkers, such as PCT and presepsin, in the assessment of odontogenic infections. In contrast to established markers such as CRP, evidence supporting these novel biomarkers remains limited [36, 37].

Within our analyses, in addition to CRP, PCT also demonstrated potential utility as a prognostic marker, whereas leukocyte concentration showed no significant association. These findings suggest that PCT may serve as a useful prognostic marker for predicting LOS in patients with odontogenic infections.

In this regard, LOS differed in various PCT subgroups. Thus, patients with PCT concentrations > 0.5 µg/l remained hospitalized almost twice as long as patients with concentrations < 0.1 µg/l with regard to the median LOS. Results of our linear and tree-based analyses also supported the association between higher PCT concentration and a prolonged LOS.

In addition to the initial blood sample, changes in inflammatory parameters during treatment are also used to make decisions in everyday clinical practice. Therefore, the relationship between changes in blood values and LOS was also analysed in our study. In this regard, no association was found between the changes in PCT, CRP and leukocyte concentrations and LOS. This seems surprising, as a decrease in inflammatory parameters should hypothetically be associated with effective surgical drainage and antibiotic therapy, as well as an improvement in the patient’s condition. A possible explanation for this could be that the blood samples were usually taken on the first or second day after surgery, when inflammation values can be significantly influenced by the surgical procedure. Consequently, it does not seem sensible to take blood samples at this point in time. Further research is needed to determine the appropriate time to include the decrease in inflammatory parameters in the prognostic assessment and to avoid unnecessary costs associated with blood sampling.

With regard to the clinical relevance of our findings, our decision tree model identified three patient subgroups with median LOS of 4, 6, and 10 days. Although these absolute differences may appear modest at first glance, even a reduction of one to two inpatient days can be clinically meaningful by reducing hospital-associated risks and facilitating earlier recovery. In addition, these differences carry economic relevance within the German DRG reimbursement system. For submandibular abscess treatment, the lower and upper LOS thresholds are 1 and 7 days, respectively, with hospital stays beyond these thresholds being associated with financial losses. Early identification of patients at risk for extended hospitalization may therefore improve both patient management and resource utilization.

When interpreting our findings, several limitations should be considered. In particular, the relatively small sample size of 61 patients limits the robustness of the statistical analyses and carries a potential risk of overfitting. This reduces the generalisability of the results to other patient populations and therefore requires external validation. This limitation applies in particular to the tree-based model, which showed considerable variation in our bootstrap analyses. Although these thresholds are consistent with established laboratory recommendations [38, 39], their application in the context of odontogenic infections still requires further validation. With regard to the transferability of our findings, it should also be noted that the study was conducted during the COVID-19 pandemic. As clinical care pathways differed substantially during this period, these circumstances may have influenced both the patient cohort as well as admission and discharge practices. This could represent an additional confounding factor affecting LOS and might limit the applicability of our findings to non-pandemic healthcare settings. It should also be noted that further multivariable analyses are required to confirm the value of PCT as an independent prognostic parameter, as LOS may additionally be influenced by various medical and non-medical confounding factors. Furthermore, the retrospective study design represents an important limitation. As a result, discharge criteria could not be prospectively standardised or systematically documented. Future prospective randomized studies are therefore warranted to further investigate the role of PCT as an independent prognostic marker in odontogenic infections.

However, our findings indicate that PCT may represent a potentially useful prognostic marker in odontogenic infections and provide a preliminary decision tree model that could support the assessment of LOS in affected patients.

## Conclusions

Elevated PCT concentrations were associated with a longer LOS, suggesting that PCT may represent a potential additional prognostic marker alongside established inflammatory parameters such as CRP in patients with odontogenic infections. Furthermore, the combined assessment of CRP and PCT may contribute to the identification of distinct LOS subgroups and provide additional value for prognostic assessment. The proposed decision tree model may represent a potential approach for clinical risk stratification, although further validation is required before its implementation in routine clinical practice. 

## Supplementary Information

Below is the link to the electronic supplementary material.

Supplementary Material 1

Supplementary Material 2

Supplementary Material 3

## Data Availability

Data are contained within the article and its Supplementary Material.

## Declarations

### Clinical trial number

The project was registered with the German Clinical Trials Register (DRKS) under registration number DRKS00039552 in March 2026.

## References

[1] Zirk M, Buller J, Goeddertz P, Rothamel D, Dreiseidler T, Zoller JE et al (2016) Empiric systemic antibiotics for hospitalized patients with severe odontogenic infections. J Craniomaxillofac Surg 44(8):1081–1088. 10.1016/j.jcms.2016.05.019 27369813 10.1016/j.jcms.2016.05.019

[2] Siqueira JF Jr., Rocas IN (2013) Microbiology and treatment of acute apical abscesses. Clin Microbiol Rev 26(2):255–273. 10.1128/CMR.00082-12 23554416 10.1128/CMR.00082-12 PMC3623375

[3] Thol F, Warwas FB, Stoll P, Sieber MA, Kramer F-J, Heim N (2025) Severe Cases of Odontogenic Abscesses, Next-Generation Sequencing Searching for Superpathogens. Case Series and Review of Current Literature. J Oral Maxillofac Surg. 10.1007/s12663-025-02850-4

[4] Bali RK, Sharma P, Gaba S, Kaur A, Ghanghas P (2015) A review of complications of odontogenic infections. Natl J Maxillofac Surg 6(2):136–143. 10.4103/0975-5950.183867 27390486 10.4103/0975-5950.183867 PMC4922222

[5] Eisler L, Wearda K, Romatoski K, Odland RM (2013) Morbidity and cost of odontogenic infections. Otolaryngol Head Neck Surg 149(1):84–88. 10.1177/0194599813485210 23585157 10.1177/0194599813485210

[6] Cachovan G, Phark JH, Schon G, Pohlenz P, Platzer U (2013) Odontogenic infections: an 8-year epidemiologic analysis in a dental emergency outpatient care unit. Acta Odontol Scand 71(3–4):518–524. 10.3109/00016357.2012.696694 22816380 10.3109/00016357.2012.696694

[7] Gams K, Shewale J, Demian N, Khalil K, Banki F (2017) Characteristics, length of stay, and hospital bills associated with severe odontogenic infections in Houston, TX. J Am Dent Assoc 148(4):221–229. 10.1016/j.adaj.2016.11.033 28129825 10.1016/j.adaj.2016.11.033

[8] Kim MK, Nalliah RP, Lee MK, Allareddy V (2012) Factors associated with length of stay and hospital charges for patients hospitalized with mouth cellulitis. Oral Surg Oral Med Oral Pathol Oral Radiol 113(1):21–28. 10.1016/j.tripleo.2011.01.012 22677688 10.1016/j.tripleo.2011.01.012

[9] Opitz D, Camerer C, Camerer DM, Raguse JD, Menneking H, Hoffmeister B et al (2015) Incidence and management of severe odontogenic infections-a retrospective analysis from 2004 to 2011. J Craniomaxillofac Surg 43(2):285–289. 10.1016/j.jcms.2014.12.002 25555896 10.1016/j.jcms.2014.12.002

[10] Qian Y, Ge Q, Zuo W, Cheng X, Xing D, Yang J et al (2021) Maxillofacial space infection experience and risk factors: a retrospective study of 222 cases. Ir J Med Sci 190(3):1045–1053. 10.1007/s11845-020-02431-z 33188628 10.1007/s11845-020-02431-z

[11] Zamiri B, Hashemi SB, Hashemi SH, Rafiee Z, Ehsani S (2012) Prevalence of Odontogenic Deep Head and Neck Spaces Infection and its Correlation with Length of Hospital Stay. J Dent 13(1):29–35

[12] Kutz A, Briel M, Christ-Crain M, Stolz D, Bouadma L, Wolff M et al (2015) Prognostic value of procalcitonin in respiratory tract infections across clinical settings. Crit Care 19(1):74. 10.1186/s13054-015-0792-1 25887979 10.1186/s13054-015-0792-1 PMC4383063

[13] Yunus I, Fasih A, Wang Y (2018) The use of procalcitonin in the determination of severity of sepsis, patient outcomes and infection characteristics. PLoS ONE 13(11):e0206527. 10.1371/journal.pone.0206527 30427887 10.1371/journal.pone.0206527 PMC6235293

[14] Nijakowski K, Ksel S, Marushko O, Nowak A, Jankowski J, Kwiatkowski J et al (2025) Impact of the COVID-19 Pandemic on Odontogenic Abscess Clinical Patterns and Predictive Factors: A Retrospective Cross-Sectional Study. J Clin Med 14(19). 10.3390/jcm14196953 10.3390/jcm14196953 PMC12524894 41096033

[15] Kumaran Geetha N, Nair I, Suraj A, Arunkumar AM (2026) Serum Biomarkers in Fascial Space Infections of Odontogenic Origin and Their Role in Prognosis of Sepsis: A Systematic Review. J Maxillofac Oral Surg 25(3):818–823. 10.1007/s12663-024-02159-8 42206107 10.1007/s12663-024-02159-8 PMC13201832

[16] Begue L, Schlund M, Raoul G, Ferri J, Lauwers L, Nicot R (2022) Biological factors predicting the length of hospital stay in odontogenic cellulitis. J Stomatol Oral Maxillofac Surg 123(3):303–308. 10.1016/j.jormas.2021.07.007 34260983 10.1016/j.jormas.2021.07.007

[17] Spuck N, Schmid M, Heim N, Klarmann-Schulz U, Hörauf A, Berger M (2022) Flexible tree-structured regression models for discrete event times. Stat Comput 33(1). 10.1007/s11222-022-10196-x

[18] Breiman L, Friedman JH, Olshen RA, Stone CJ (2000) Classification and Regression Trees

[19] Schwarz G (1978) Estimating the Dimension of a Model. Ann Stat 6:461–464

[20] Tutz G, Schmid M (2016) Modeling Discrete Time-to-Event Data. 10.1007/978-3-319-28158-2

[21] Berger M, Schmid M (2018) Semiparametric regression for discrete time-to-event data. Stat Modelling 18(3–4):322–345. 10.1177/1471082x17748084

[22] Sanchez R, Mirada E, Arias J, Pano JR, Burgueno M (2011) Severe odontogenic infections: epidemiological, microbiological and therapeutic factors. Med Oral Patol Oral Cir Bucal 16(5):e670–e676. 10.4317/medoral.16995 20711116 10.4317/medoral.16995

[23] Thol F, Warwas FB, Spuck N, Kramer FJ, Heim N (2024) Microbial spectrum and resistance of odontogenic abscesses - microbiological analysis using next generation sequencing. Clin Oral Investig 29(1):8. 10.1007/s00784-024-06097-0 39656293 10.1007/s00784-024-06097-0 PMC11631990

[24] Warwas FB, Thol F, Sieber MA, Spuck N, Kramer FJ, Heim N (2025) Next-generation sequencing improves pathogen identification in odontogenic abscesses, could this affect clinical outcomes? Clin Oral Investig 29(9):430. 10.1007/s00784-025-06504-0 40892172 10.1007/s00784-025-06504-0 PMC12405399

[25] Heim N, Faron A, Wiedemeyer V, Reich R, Martini M (2017) Microbiology and antibiotic sensitivity of head and neck space infections of odontogenic origin. Differences in inpatient and outpatient management. J Craniomaxillofac Surg 45(10):1731–1735. 10.1016/j.jcms.2017.07.013 28838838 10.1016/j.jcms.2017.07.013

[26] Al-Nawas BK (2016) J.: S3-Leitlinie (Langversion): Odontogene Infektionen. https://register.awmf.org/assets/guidelines/007-006l_S3_Odontogene_Infektionen_2017-12-abgelaufen.pdf Accessed 7 February 2024

[27] Boscolo-Rizzo P, Da Mosto MC (2009) Submandibular space infection: a potentially lethal infection. Int J Infect Dis 13(3):327–333. 10.1016/j.ijid.2008.07.007 18952475 10.1016/j.ijid.2008.07.007

[28] Azenha MR, Homsi G, Garcia IR (2012) Jr. Multiple brain abscess from dental origin: case report and literature review. Oral Maxillofac Surg 16(4):393–397. 10.1007/s10006-011-0308-3 22160491 10.1007/s10006-011-0308-3

[29] Pappa H, Jones DC (2005) Mediastinitis from odontogenic infection. A case report. Br Dent J 198(9):547–548. 10.1038/sj.bdj.4812302 15895047 10.1038/sj.bdj.4812302

[30] Pham Dang N, Delbet-Dupas C, Mulliez A, Devoize L, Dallel R, Barthelemy I (2020) Five Predictors Affecting the Prognosis of Patients with Severe Odontogenic Infections. Int J Environ Res Public Health 17(23). 10.3390/ijerph17238917 10.3390/ijerph17238917 PMC7730806 33266250

[31] Bloomquist JNJ, Brown R, Looney K, Walker S, Day D (2025) Associations of time to the operating room on outcomes in odontogenic infection. BMC Oral Health 25(1):108. 10.1186/s12903-024-05300-8 39833836 10.1186/s12903-024-05300-8 PMC11749400

[32] Heim N, Wiedemeyer V, Reich RH, Martini M (2018) The role of C-reactive protein and white blood cell count in the prediction of length of stay in hospital and severity of odontogenic abscess. J Craniomaxillofac Surg 46(12):2220–2226. 10.1016/j.jcms.2018.10.013 30416035 10.1016/j.jcms.2018.10.013

[33] Kaercher D, Thelen P, Ruettermann M, Li L, Hamprecht A (2024) Outcome predictors of odontogenic abscesses in the elderly. Front Oral Health 5:1486182. 10.3389/froh.2024.1486182 39687480 10.3389/froh.2024.1486182 PMC11646891

[34] Sakkas A, Rana M, Scheurer M, Kasper R, Ebeling M, Wilde F et al (2025) Factors influencing recurrence after in-hospital treatment of odontogenic-related oromaxillofacial infections: a single-centre experience. BMC Oral Health 25(1):1273. 10.1186/s12903-025-06662-3 40721760 10.1186/s12903-025-06662-3 PMC12305926

[35] Kang SH, Kim MK (2019) Antibiotic sensitivity and resistance of bacteria from odontogenic maxillofacial abscesses. J Korean Assoc Oral Maxillofac Surg 45(6):324–331. 10.5125/jkaoms.2019.45.6.324 31966977 10.5125/jkaoms.2019.45.6.324 PMC6955427

[36] Kang ES, Lee JH (2022) Diagnostic value of presepsin in odontogenic infection: a retrospective study. Maxillofac Plast Reconstr Surg 44(1):22. 10.1186/s40902-022-00353-7 35666350 10.1186/s40902-022-00353-7 PMC9170860

[37] Martinovic D, Puizina E, Kos B, Puizina J, Jurina L, Martinovic L et al (2026) Dynamics of Inflammatory Parameters in Hospitalized and Surgically Treated Patients with Odontogenic Abscesses. Med (Kaunas) 62(4). 10.3390/medicina62040614 10.3390/medicina62040614 PMC13117892 42075488

[38] Samsudin I, Vasikaran SD (2017) Clinical Utility and Measurement of Procalcitonin. Clin Biochem Rev 38(2):59–68 29332972 PMC5759088

[39] Horns H, Draenert R, Nistal M (2021) [Procalcitonin]. MMW Fortschr Med 163(11):54–55. 10.1007/s15006-021-9959-7 34086237 10.1007/s15006-021-9959-7 PMC8175922

