# Supplementary material for: Procalcitonin as a predictor of length of stay in patients with odontogenic infections
Source: Oral Maxillofac Surg. 2026 Aug 7;30(1):128. doi: 10.1007/s10006-026-01617-6 (PMC13451498; doi:10.1007/s10006-026-01617-6)
Supplement: Supplementary file 1 [file 10006_2026_1617_MOESM1_ESM.pdf]

UNIVERSITÄT BONN

ukb universitäts klinikumbonn

Geschäftsstelle Ethikkommission, Medizinische Fakultät,
Venusberg-Campus 1, 53127 Bonn

Herr
Dr. med. Felix Thol
Klinik für Mund-, Kiefer- und plastische Gesichtschirurgie
Universitätsklinikum Bonn
Venusberg-Campus 1
53127 Bonn

Ethikkommission

Prof. Dr. med. Kurt Racké
Vorsitzender

Geschäftsstelle

Geschäftsführerin
Ass. iur. Anke Stümpfig
Telefon: +49 228 287 10418

Ass. der Geschäftsführung
Michaela Hubert
Telefon +49 228 287 51931
Fax +49 228 287 9080206

Wiss. Referent*innen
Dr. rer. nat. Daniel Breitkopf
Telefon: +49 228 287 51930

Dr. rer. nat. Anke Gertzmann
Telefon: +49 228 287 51563

Dr. rer. nat. Anja Wieland
Telefon: +49 228 287 51422

Dr. rer. nat. Almut Wingen
Telefon +49 228 287 51564

Sachbearbeitung
Anja Dorschner
Telefon +49 228 287 51561

Michaela Hubert
Telefon +49 228 287 51931

Martina Niesen
Telefon +49 228 287 51938

Bettina Roßbach
Telefon +49 228 287 51282

Claudia Schmidt
Telefon +49 228 287 51562

ethik@ukbonn.de

http://ethik.meb.uni-bonn.de

Postanschrift
Geschäftsstelle der Ethikkommission
Medizinische Fakultät
Venusberg-Campus 1
Gebäude 74 / 4. OG
53127 Bonn

Telefonsprechzeiten
Montag-Donnerstag
9:00-12:00 Uhr

Datum: 24.03.2026
KRa/Hu/Wie

Aktenzeichen: 2026-122-BO (bitte stets angeben!)
<u>Retrospektive Datenanalyse</u>

Studienleitung:	Dr. med. Felix Thol, Klinik für Mund-, Kiefer- und plastische Gesichtschirurgie
Studientitel:	Procalcitonin als Prädiktor für die Aufenthaltsdauer von Patienten mit odontogenen Infektionen

Auflistung der eingereichten Unterlagen siehe Anhang
Hier: Eingang in ethikPool am 03.03.2026/ 08.03.2026

Sehr geehrter Herr Dr. Thol,

die Ethikkommission für klinische Versuche am Menschen und epidemiologische Forschung mit personenbezogenen Daten der Medizinischen Fakultät der Rheinischen Friedrich-Wilhelms-Universität Bonn hat in ihrer Sitzung vom 23.03.2026 das oben genannte Forschungsvorhaben beraten.

Auf der Grundlage der übersandten Unterlagen und dem derzeitigen Informationsstand erhebt die Ethikkommission keine ethischen oder rechtlichen Einwände gegen die Durchführung des oben genannten Forschungsvorhabens,

gibt aber folgenden Hinweis.
Ethikantrag_PCT_V2.pdf

1) S. 2: Die Angabe zur Studienregistrierung ist verwirrend, da laut Anschreiben und im Abschnitt zum Datenschutz eine Registrierung im DRKS erfolgt ist.

Alle relevanten Änderungen des Forschungsvorhabens sind der Ethikkommission entsprechend der maßgebenden Deklaration des Weltärztebundes von Helsinki in der letzten revidierten Fassung zur erneuten Beratung vorzulegen.

Bitte teilen Sie der Ethikkommission das Ende des Forschungsvorhabens mit und reichen Sie einen kurzen Ergebnisbericht/Publikation ein.

Die Ethikkommission wünscht Ihnen viel Erfolg.

Mit freundlichen Grüßen

Prof. Dr. Kurt Racké
Vorsitzender der Ethikkommission

Allgemeine Hinweise:

- Die ethische und rechtliche Verantwortung für das Forschungsvorhaben und seine Durchführung verbleibt uneingeschränkt bei der/den für das Forschungsvorhaben verantwortlichen Person/en und wird nicht von der Ethikkommission übernommen.
- Sollte das Forschungsvorhaben nicht nach Ablauf von 5 Jahren nach Erteilung des Votums abgeschlossen sein, bittet die Ethikkommission schon jetzt um einen Zwischenbericht zum Forschungsvorhaben, um prüfen zu können, ob es einer weiteren berufsrechtlichen Beratung bedarf. Dieser Zwischenbericht ist ohne weitergehende Aufforderung an die Geschäftsstelle zu schicken.
- Die Ethikkommission geht davon aus, dass dieses Forschungsvorhaben gemäß den Prinzipien des Weltärztebundes, niedergelegt in der Deklaration von Helsinki und ggf. einschlägigen Rechtsvorschriften, durchgeführt wird.
- Datenschutzrechtliche Aspekte von Forschungsvorhaben werden durch die Ethikkommission grundsätzlich nur kursorisch geprüft. Diese zustimmende Bewertung ersetzt mithin nicht die Konsultation des jeweils zuständigen Datenschutzbeauftragten durch die jeweils verantwortliche Person.
- Die Ethikkommission der Medizinischen Fakultät der Rheinischen Friedrich-Wilhelms-Universität Bonn arbeitet gemäß den nationalen gesetzlichen Bestimmungen und den ICH-GCP Richtlinien. Den Beratungen der Ethikkommission der Medizinischen Fakultät der Rheinischen Friedrich-Wilhelms-Universität Bonn liegt gemäß der gültigen Berufsordnung die maßgebende Deklaration des Weltärztebundes von Helsinki in der letzten revidierten Fassung zugrunde.

Auflistung der eingereichten Unterlagen

Eingang in ethikPool am 08.03.2026

Anschreiben_FT.pdf
Ethikantrag_PCT_V2.pdf

Eingang in ethikPool am 03.03.2026:

Anschreiben_Ethikkommission_PCT_ FT.pdf
Antragsformular_FT.pdf
Studienprotokoll_PCT_FT.pdf
Unterschriftenseite_FT.pdf
Eignung des Studienzentrums.pdf
Finanzierung.pdf
