# Supplementary material for: Procalcitonin as a predictor of length of stay in patients with odontogenic infections
Source: Oral Maxillofac Surg. 2026 Aug 7;30(1):128. doi: 10.1007/s10006-026-01617-6 (PMC13451498; doi:10.1007/s10006-026-01617-6)
Supplement: Supplementary file 2 [file 10006_2026_1617_MOESM2_ESM.pdf]

Berufsordnung
für die nordrheinischen Ärztinnen und Ärzte

www.aekno.de

Die Kammerversammlung der Ärztekammer Nordrhein hat in ihrer Sitzung am 22. März 2025 aufgrund § 31 des Heilberufsgesetzes vom 9. Mai 2000 (GV. NRW. S. 403), zuletzt geändert durch Gesetz vom 30. Januar 2024 (GV. NRW. S. 75) folgende Änderung der Berufsordnung für die nordrheinischen Ärztinnen und Ärzte vom 14. November 1998 (MBl. NRW. 1999 S. 350), zuletzt geändert am 19. November 2019 (MBl. NRW. 2020 S. 182) beschlossen.

Ärztekammer Nordrhein
Körperschaft des öffentlichen Rechts

Berufsordnung
für die nordrheinischen Ärztinnen und Ärzte
vom 14.11.1998
in der Fassung vom 22.03.2025

(in Kraft getreten am 23.07.2025)

Inhaltsübersicht

A. Präambel

B. Regeln zur Berufsausübung

I. Grundsätze

§ 1 Ärztliche Aufgaben
§ 2 Allgemeine ärztliche Berufspflichten
§ 3 Unvereinbarkeiten
§ 4 Fortbildung
§ 5 Qualitätssicherung
§ 6 Mitteilung von unerwünschten Arzneimittelwirkungen

II. Pflichten gegenüber Patientinnen und Patienten

§ 7 Behandlungsgrundsätze und Verhaltensregeln
§ 8 Aufklärungspflicht
§ 9 Schweigepflicht
§ 10 Dokumentationspflicht
§ 11 Ärztliche Untersuchungs- und Behandlungsmethoden
§ 12 Honorar und Vergütungsabsprachen

III. Besondere medizinische Verfahren und Forschung

§ 13 Besondere medizinische Verfahren
§ 14 Erhaltung des ungeborenen Lebens und Schwangerschaftsabbruch
§ 15 Forschung
§ 16 Beistand für Sterbende

IV. Berufliches Verhalten

1. Berufsausübung

§ 17 Niederlassung und Ausübung der Praxis
§ 18 Berufliche Kooperation
§ 18 a Ankündigung von Berufsausübungsgemeinschaften und sonstigen Kooperationen
§ 19 Beschäftigung angestellter Praxisärztinnen und -ärzte
§ 20 Vertretung
§ 21 Haftpflichtversicherung
§ 22 (unbesetzt)
§ 22 a (unbesetzt)
§ 23 Ärztinnen und Ärzte im Beschäftigungsverhältnis
§ 23 a Kooperationsgemeinschaft zwischen Ärzten und Angehörigen anderer Fachberufe im Gesundheitswesen
§ 23 b (aufgehoben)
§ 23 c Praxisverbund
§ 24 Verträge über ärztliche Tätigkeit
§ 25 Ärztliche Gutachten und Zeugnisse
§ 26 Ärztlicher Notfalldienst

2. Berufliche Kommunikation

§ 27 Erlaubte Information und berufswidrige Werbung
§ 28 (aufgehoben)

3. Berufliche ärztliche Zusammenarbeit

§ 29 Kollegiale Zusammenarbeit
§ 29a Zusammenarbeit mit Dritten

4. Wahrung der ärztlichen Unabhängigkeit bei der Zusammenarbeit mit Dritten

§ 30 Ärztliche Unabhängigkeit
§ 31 Unerlaubte Zuweisung
§ 32 Unerlaubte Zuwendungen
§ 33 Zuwendungen bei vertraglicher Zusammenarbeit
§ 34 (aufgehoben)
§ 35 (aufgehoben)

C. Verhaltensregeln (Grundsätze korrekter ärztlicher Berufsausübung)

Nr. 1 (aufgehoben)
Nr. 2 (aufgehoben)
Nr. 3 (aufgehoben)

D. Ergänzende Bestimmungen zu einzelnen ärztlichen Berufpflichten

I. Pflichten bei grenzüberschreitender ärztlicher Tätigkeit

Nr. 1 (aufgehoben)
Nr. 2 (aufgehoben)

II. Pflichten in besonderen medizinischen Situationen

Nr. 3 (aufgehoben)
Nr. 4 (aufgehoben)

D. **Anlage-** Richtlinie zur Durchführung der assistierten Reproduktion gemäß § 13 Berufsordnung für die nordrheinischen Ärztinnen und Ärzte

(aufgehoben)

Gelöbnis

Als Mitglied der ärztlichen Profession gelobe ich feierlich, mein Leben in den Dienst der Menschlichkeit zu stellen. Die Gesundheit und das Wohlergehen meiner Patientin oder meines Patienten werden mein oberstes Anliegen sein.

Ich werde die Autonomie und die Würde meiner Patientin oder meines Patienten respektieren.

Ich werde den höchsten Respekt vor menschlichem Leben wahren.

Ich werde nicht zulassen, dass Erwägungen zu Alter, Krankheit oder Behinderung, Glaube, ethnischer Herkunft, Geschlecht, Staatsangehörigkeit, politischer Zugehörigkeit, Rasse, sexueller Orientierung, sozialer Stellung oder jegliche andere Erwägungen mich von der Erfüllung meiner Pflichten gegenüber meiner Patientin oder meinem Patienten abbringen.

Ich werde die mir anvertrauten Geheimnisse auch über den Tod der Patientin oder des Patienten hinaus wahren.

Ich werde meinen Beruf nach bestem Wissen und Gewissen, mit Würde und im Einklang mit guter medizinischer Praxis ausüben.

Ich werde die Ehre und die edlen Traditionen des ärztlichen Berufes fördern.

Ich werde meinen Lehrerinnen und Lehrern, meinen Kolleginnen und Kollegen und meinen Schülerinnen und Schülern die ihnen gebührende Achtung und Dankbarkeit erweisen.

Ich werde mein medizinisches Wissen zum Wohle der Patientin oder des Patienten und zur Verbesserung der Gesundheitsversorgung teilen.

Ich werde auf meine eigene Gesundheit, mein Wohlergehen und meine Fähigkeiten achten, um eine Behandlung auf höchstem Niveau leisten zu können.

Ich werde, selbst unter Bedrohung, mein medizinisches Wissen nicht zur Verletzung von Menschenrechten und bürgerlichen Freiheiten anwenden.

Ich gelobe dies feierlich, aus freien Stücken und bei meiner Ehre.

A. Präambel

Die auf der Grundlage der Kammer- und Heilberufsgesetze beschlossene Berufsordnung stellt die Überzeugung der Ärzteschaft zum Verhalten von Ärztinnen und Ärzten gegenüber ihren Patientinnen und Patienten, den Kolleginnen und Kollegen, den anderen Partnerinnen und Partnern im Gesundheitswesen sowie zum Verhalten in der Öffentlichkeit dar. Dafür geben sich die in Nordrhein tätigen Ärztinnen und Ärzte die nachstehende Berufsordnung. Mit der Festlegung von Berufspflichten der Ärztinnen und Ärzte dient die Berufsordnung zugleich dem Ziel,

- das Vertrauen zwischen Ärztinnen und Ärzten und ihren Patientinnen und Patienten zu erhalten und zu fördern;

- die Qualität der ärztlichen Tätigkeit im Interesse der Gesundheit der Bevölkerung sicherzustellen;

- die Freiheit und das Ansehen des Arztberufes zu wahren;

- berufswürdiges Verhalten zu fördern und berufsunwürdiges Verhalten zu verhindern.

B. Regeln zur Berufsausübung

I. Grundsätze

§ 1

Ärztliche Aufgaben

(1) Ärztinnen und Ärzte dienen der Gesundheit des einzelnen Menschen und der Bevölkerung. Der ärztliche Beruf ist kein Gewerbe. Er ist seiner Natur nach ein freier Beruf.

(2) Ärztliche Aufgabe ist es, das Leben zu erhalten, die Gesundheit zu schützen und wiederherzustellen, Leiden zu lindern, Sterbenden Beistand zu leisten und an der Erhaltung der natürlichen Lebensgrundlagen im Hinblick auf ihre Bedeutung für die Gesundheit der Menschen mitzuwirken.

§ 2

Allgemeine ärztliche Berufspflichten

(1) Ärztinnen und Ärzte üben ihren Beruf nach ihrem Gewissen, den Geboten der ärztlichen Ethik und der Menschlichkeit aus. Sie dürfen keine Grundsätze anerkennen und keine Vorschriften oder Anweisungen beachten, die mit dieser Aufgabe nicht vereinbar sind oder deren Befolgung sie nicht verantworten können.

(2) Ärztinnen und Ärzte haben ihren Beruf gewissenhaft auszuüben und dem ihnen bei ihrer Berufsausübung entgegengebrachten Vertrauen zu entsprechen. Sie haben dabei ihr ärztliches Handeln am Wohl der Patientinnen und Patienten auszurichten. Insbesondere dürfen sie nicht das Interesse Dritter über das Wohl der Patientinnen und Patienten stellen.

(3) Eine gewissenhafte Ausübung des Berufs erfordert insbesondere die notwendige fachliche Qualifikation und die Beachtung des anerkannten Standes der medizinischen Erkenntnisse.

(4) Ärztinnen und Ärzte dürfen hinsichtlich ihrer ärztlichen Entscheidungen keine Weisungen von Nichtärzten entgegennehmen.

(5) Ärztinnen und Ärzte sind verpflichtet, die für die Berufsausübung geltenden Vorschriften zu beachten.

(6) Unbeschadet der in den nachfolgenden Vorschriften geregelten besonderen Auskunfts- und Anzeigepflichten haben Ärztinnen und Ärzte auf Anfragen der Ärztekammer, welche diese zur Erfüllung ihrer gesetzlichen Aufgaben bei der Berufsaufsicht an sie richtet, in angemessener Frist zu antworten und auf Verlangen Nachweise zu erbringen.

(7) Werden Ärztinnen und Ärzte, die in einem anderen Mitgliedstaat der Europäischen Union niedergelassen sind oder dort ihre berufliche Tätigkeit entfalten, vorübergehend und gelegentlich im Geltungsbereich dieser Berufsordnung grenzüberschreitend ärztlich tätig, ohne eine Niederlassung zu begründen, so haben sie die Vorschriften dieser Berufsordnung zu beachten.

§ 3

Unvereinbarkeiten

(1) Ärztinnen und Ärzten ist neben der Ausübung ihres Berufes die Ausübung einer anderen Tätigkeit untersagt, welche mit den ethischen Grundsätzen des ärztlichen Berufes nicht vereinbar ist. Ihnen ist auch verboten, ihren Namen in Verbindung mit einer ärztlichen Berufsbezeichnung in unlauterer Weise für gewerbliche Zwecke herzugeben. Ebenso wenig dürfen sie zulassen, dass von ihrem Namen oder von ihrem beruflichen Ansehen in solcher Weise Gebrauch gemacht wird.

(2) Ärztinnen und Ärzten ist untersagt, im Zusammenhang mit der Ausübung ihrer ärztlichen Tätigkeit Waren und andere Gegenstände abzugeben oder unter ihrer Mitwirkung abgeben zu lassen sowie gewerbliche Dienstleistungen zu erbringen oder erbringen zu lassen, soweit nicht die Abgabe des Produkts oder die Dienstleistung wegen ihrer Besonderheiten notwendiger Bestandteil der Therapie sind.

§ 4

Fortbildung

(1) Ärztinnen und Ärzte, die ihren Beruf ausüben, sind verpflichtet, sich in dem Umfang beruflich fortzubilden, wie es zur Erhaltung und Entwicklung der zu ihrer Berufsausübung erforderlichen Fachkenntnisse notwendig ist.

(2) Auf Verlangen müssen Ärztinnen und Ärzte ihre Fortbildung nach Absatz 1 gegenüber der Ärztekammer durch ein Fortbildungszertifikat einer Ärztekammer nachweisen.

§ 5

Qualitätssicherung

(1) Ärztinnen und Ärzte sind verpflichtet, an den von der Ärztekammer eingeführten Maßnahmen zur Sicherung der Qualität der ärztlichen Tätigkeit teilzunehmen und der Ärztekammer die hierzu erforderlichen Auskünfte zu erteilen.

(2) Bei besonderen medizinischen Verfahren nach § 13 Absatz 1 haben Ärztinnen und Ärzte an Qualitätssicherungsmaßnahmen teilzunehmen, in der die Behandlungsqualität unter Struktur- und Prozessgesichtspunkten verfahrens- und ergebnisorientiert geprüft und anlassbezogen beraten wird.

(3) Bei Verfahren und Maßnahmen der assistierten Befruchtung nach § 13 Absatz 3 haben Ärztinnen und Ärzte an gesondert eingeführten besonderen Qualitätssicherungsverfahren teilzunehmen und die hierzu erforderlichen Auskünfte zu erteilen und Nachweise zu erbringen, insbesondere jährlich eine EDV-gestützte Dokumentation an die von der Ärztekammer für die Datenannahme bestimmte zuständige Stelle zu übermitteln. Die Datenerfassung hat den Anforderungen an Prospektivität zu entsprechen, die dadurch zu gewährleisten ist, dass die Angaben zum Behandlungszyklus innerhalb von acht Tagen nach Beginn der hormonellen Stimulation eingegeben werden.

§ 6

Mitteilung von unerwünschten Arzneimittelwirkungen

Ärztinnen und Ärzte sind verpflichtet, die ihnen aus ihrer ärztlichen Behandlungstätigkeit bekannt werdenden unerwünschten Wirkungen von Arzneimitteln der Arzneimittelkommission der deutschen Ärzteschaft und bei Medizinprodukten auftretende Vorkommnisse der zuständigen Behörde mitzuteilen.

II. Pflichten gegenüber Patientinnen und Patienten

§ 7

Behandlungsgrundsätze und Verhaltensregeln

(1) Jede medizinische Behandlung hat unter Wahrung der Menschenwürde und unter Achtung der Persönlichkeit, des Willens und der Rechte, insbesondere des Selbstbestimmungsrechts von Patientinnen bzw. Patienten zu erfolgen. Das Recht der Patientinnen und Patienten, empfohlene Untersuchungs- und Behandlungsmaßnahmen abzulehnen, ist zu respektieren.

(2) Ärztinnen und Ärzte achten das Recht der Patientinnen und Patienten, ihre Ärztin bzw. ihren Arzt frei wählen oder wechseln zu können. Von Notfällen oder besonderen rechtlichen Verpflichtungen abgesehen, darf auch ärztlicherseits eine Behandlung abgelehnt werden. Der begründete Wunsch der Patientin oder des Patienten, eine weitere Ärztin oder einen weiteren Arzt zuzuziehen oder dorthin überwiesen zu werden, soll in der Regel nicht abgelehnt werden.

(3) Ärztinnen und Ärzte haben im Interesse der Patientinnen und Patienten mit anderen Ärztinnen und Ärzten und Angehörigen anderer Fachberufe im Gesundheitswesen zusammenzuarbeiten. Soweit dies für die Diagnostik und Therapie erforderlich ist, haben sie rechtzeitig andere Ärztinnen und Ärzte hinzuzuziehen oder ihnen die Patientin oder den Patienten zur Fortsetzung der Behandlung zu überweisen.

(4) Ärztinnen und Ärzte beraten und behandeln Patientinnen und Patienten im persönlichen Kontakt. Sie können dabei Kommunikationsmedien unterstützend einsetzen. Eine ausschließliche Beratung oder Behandlung über Kommunikationsmedien ist im Einzelfall erlaubt, wenn dies ärztlich vertretbar ist und die erforderliche ärztliche Sorgfalt insbesondere durch die Art und Weise der Befunderhebung, Beratung, Behandlung sowie Dokumentation gewahrt wird und die Patientin oder der Patient auch über die Besonderheiten der ausschließlichen Beratung und Behandlung über Kommunikationsmedien aufgeklärt wird.

(5) Angehörige von Patientinnen und Patienten und andere Personen dürfen bei der Untersuchung und der Behandlung anwesend sein, wenn die verantwortliche Ärztin bzw. der verantwortliche Arzt und die Patientin oder der Patient zustimmen.

(6) Ärztinnen und Ärzte haben Patientinnen und Patienten gebührende Aufmerksamkeit entgegenzubringen und mit Patientenkritik und Meinungsverschiedenheiten sachlich und korrekt umzugehen.

(7) Bei der Überweisung von Patientinnen und Patienten an Kolleginnen oder Kollegen oder ärztlich geleitete Einrichtungen, haben Ärztinnen und Ärzte rechtzeitig die erhobenen Befunde zu übermitteln und über die bisherige Behandlung zu informieren, soweit das Einverständnis der Patientinnen und Patienten vorliegt oder anzunehmen ist. Dies gilt insbesondere bei der Krankenhauseinweisung und -entlassung. Originalunterlagen sind zurückzugeben.

(8) Ärztinnen und Ärzte dürfen einer missbräuchlichen Verwendung ihrer Verschreibung keinen Vorschub leisten.

§ 8

Aufklärungspflicht

Zur Behandlung bedürfen Ärztinnen und Ärzte der Einwilligung der Patientin oder des Patienten. Der Einwilligung hat grundsätzlich die erforderliche Aufklärung im persönlichen Gespräch vorauszugehen. Die Aufklärung hat der Patientin oder dem Patienten insbesondere vor operativen Eingriffen Wesen, Bedeutung und Tragweite der Behandlung einschließlich Behandlungsalternativen und die mit ihr verbundenen Risiken in verständlicher und angemessener Weise zu verdeutlichen. Insbesondere vor diagnostischen oder operativen Eingriffen ist soweit möglich eine ausreichende Bedenkzeit vor der weiteren Behandlung zu gewährleisten. Je weniger eine Maßnahme medizinisch geboten oder je größer ihre Tragweite ist, umso ausführlicher und eindrücklicher sind Patientinnen oder Patienten über erreichbare Ergebnisse und Risiken aufzuklären.

§ 9

Schweigepflicht

(1) Ärztinnen und Ärzte haben über das, was ihnen in ihrer ärztlichen Eigenschaft anvertraut oder bekannt geworden ist - auch über den Tod der Patientin bzw. des Patienten hinaus - zu schweigen. Dazu gehören auch schriftliche Mitteilungen der Patientin bzw. des Patienten, ärztliche Aufzeichnungen, Röntgenaufnahmen und sonstige Untersuchungsbefunde.

(2) Ärztinnen und Ärzte sind zur Offenbarung befugt, soweit sie von der Schweigepflicht entbunden worden sind oder soweit die Offenbarung zum Schutze eines höherwertigen Rechtsgutes erforderlich ist. Gesetzliche Aussage- und Anzeigepflichten bleiben unberührt. Soweit gesetzliche Vorschriften die ärztliche Schweigepflicht einschränken, soll die Patientin oder der Patient darüber unterrichtet werden.

(3) Ärztinnen und Ärzte dürfen ihren Mitarbeiterinnen und Mitarbeitern sowie Personen, die zur Vorbereitung auf den Beruf an der ärztlichen Tätigkeit teilnehmen, Informationen über Patientinnen und Patienten zugänglich machen. Über die gesetzliche Pflicht zur Verschwiegenheit haben sie diese zu belehren und dies schriftlich festzuhalten.

(4) Gegenüber den Mitarbeiterinnen und Mitarbeitern von Dienstleistungsunternehmen sowie sonstigen Personen, die an der beruflichen Tätigkeit mitwirken, sind Ärztinnen und Ärzte zur Offenbarung befugt, soweit dies für die Inanspruchnahme der Tätigkeit der mitwirkenden Personen erforderlich ist. Ärztinnen und Ärzte haben dafür zu sorgen, dass die mitwirkenden Personen schriftlich zur Geheimhaltung verpflichtet werden. Diese Verpflichtung zur Geheimhaltung haben Ärztinnen und Ärzte vorzunehmen oder auf das von ihnen beauftragte Dienstleistungsunternehmen zu übertragen.

(5) Wenn mehrere Ärztinnen und Ärzte gleichzeitig oder nacheinander dieselbe Patientin oder denselben Patienten untersuchen oder behandeln, sind sie untereinander von der Schweigepflicht insoweit befreit, als deren Einverständnis vorliegt oder anzunehmen ist.

§ 10

Dokumentationspflicht

(1) Ärztinnen und Ärzte haben über die in Ausübung ihres Berufes gemachten Feststellungen und getroffenen Maßnahmen die erforderlichen Aufzeichnungen zu machen. Diese sind nicht nur ärztliche Gedächtnisstützen, sie dienen auch dem Interesse der Patientin oder des Patienten an einer ordnungsgemäßen Dokumentation.

(2) Ärztinnen und Ärzte haben Patientinnen und Patienten auf deren Verlangen in die sie betreffende Dokumentation Einsicht zu gewähren, soweit der Einsichtnahme nicht erhebliche therapeutische Gründe oder erhebliche Rechte der Ärztin, des Arztes oder Dritter entgegenstehen. Auf Verlangen sind ihnen Kopien der Unterlagen gegen Erstattung der Kosten herauszugeben.

(3) Ärztliche Aufzeichnungen sind für die Dauer von zehn Jahren nach Abschluss der Behandlung aufzubewahren, soweit nicht nach gesetzlichen Vorschriften eine längere Aufbewahrungspflicht besteht.

(4) Nach Aufgabe der Praxis haben Ärztinnen und Ärzte ihre ärztlichen Aufzeichnungen und Untersuchungsbefunde gem. Abs. 3 aufzubewahren oder dafür Sorge zu tragen, dass sie in gehörige Obhut gegeben werden. Ärztinnen und Ärzte, denen bei einer Praxisaufgabe oder Praxisübergabe ärztliche Aufzeichnungen über Patientinnen und Patienten in Obhut gegeben werden, müssen diese Aufzeichnungen unter Verschluss halten und dürfen sie nur mit deren Einwilligung einsehen oder weitergeben.

(5) Aufzeichnungen auf elektronischen Datenträgern oder anderen Speichermedien bedürfen besonderer Sicherungs- und Schutzmaßnahmen, um deren Veränderung, Vernichtung oder unrechtmäßige Verwendung zu verhindern.

(6) Ärztinnen und Ärzte dürfen Angaben zur Approbation oder Erlaubnis zur vorübergehenden Ausübung des ärztlichen Berufes sowie zu Bezeichnungen nach der Weiterbildungsordnung nur durch eine von der Ärztekammer betriebene oder mit der Ärztekammer durch einen Kooperationsvertrag verbundene Zertifizierungsstelle ”Schlüssel-Zertifikate” oder ”Attribute-Zertifikate” aufnehmen lassen.

§ 11

Ärztliche Untersuchungs- und Behandlungsmethoden

(1) Mit Übernahme der Behandlung verpflichten sich Ärztinnen und Ärzte gegenüber ihren Patientinnen und Patienten zur gewissenhaften Versorgung mit geeigneten Untersuchungs- und Behandlungsmethoden.

(2) Der ärztliche Berufsauftrag verbietet es, diagnostische oder therapeutische Methoden unter missbräuchlicher Ausnutzung des Vertrauens, der Unwissenheit, der Leichtgläubigkeit oder der Hilflosigkeit von Patientinnen und Patienten anzuwenden. Unzulässig ist es auch, Heilerfolge, insbesondere bei nicht heilbaren Krankheiten als gewiss zuzusichern.

§ 12

Honorar und Vergütungsabsprachen

(1) Die Honorarforderung muss angemessen sein. Für die Bemessung ist die amtliche Gebührenordnung (GOÄ) die Grundlage, soweit nicht andere gesetzliche Vergütungsregelungen gelten. Die Sätze nach der GOÄ dürfen nicht in unlauterer Weise unterschritten werden.

(2) Die Übermittlung von Daten an Dritte zum Zweck der privatärztlichen Abrechnung ist nur zulässig, wenn die Patientin oder der Patient in die Übermittlung der für die Abrechnung erforderlichen Daten nachweisbar eingewilligt hat.

(3) Ärztinnen und Ärzte können Verwandten, Kolleginnen und Kollegen, deren Angehörigen, mittellosen Patientinnen und Patienten das Honorar ganz oder teilweise erlassen.

(4) Auf Antrag einer oder eines Beteiligten gibt die Ärztekammer eine gutachterliche Äußerung über die Angemessenheit der Honorarforderung ab.

III. Besondere medizinische Verfahren und Forschung

§ 13

Besondere medizinische Verfahren

(1) Soweit Ärztinnen und Ärzte medizinische Verfahren und Maßnahmen anwenden, die besondere ethische Fragestellungen aufwerfen, zu denen die Ärztekammer Maßgaben oder Empfehlungen festgelegt hat, haben sie diese anzuzeigen und nachzuweisen, dass sie die für diese Verfahren und Maßnahmen festgelegten Voraussetzungen erfüllen und beachten.

(2) Ärztinnen und Ärzte können nicht verpflichtet werden, entgegen ihrer Gewissensüberzeugung medizinische Verfahren oder Maßnahmen mit besonderer ethischer Fragestellung anzuwenden oder bei deren Anwendung mitzuwirken.

(3) Bei allen Maßnahmen der assistierten Befruchtung, bei der Keimzellen (Ei- und/oder Samenzellen) oder Embryonen verwendet werden, muss die Verwendung medizinisch indiziert und der gesundheitliche Schutz der Mutter und des Kindes gewährleistet sein. Ärztinnen und Ärzte haben die beabsichtigte Tätigkeit anzuzeigen und nachzuweisen, dass sie aufgrund ihrer Qualifikation, sachlichen und personellen Ausstattung und ihrer behördlichen Genehmigungen eine dem Stand der Wissenschaft entsprechende Durchführung der Verfahren und Maßnahmen gewährleisten. Die Verwendung von Keimzellen oder Embryonen ist zulässig bei Ehepaaren und Lebensgemeinschaften. Ärztinnen und Ärzte haben sich vor der Durchführung heterologer Verfahren und bei nicht verheirateten Paaren zu vergewissern, dass sich das Paar hat juristisch beraten lassen und eine notarielle Dokumentation erfolgt ist. Die spezifischen Dokumentations-, Auskunfts- und Aufklärungs- sowie Meldepflichten sind zu beachten (insb. Samenspenderregistergesetz).

(4) Die Durchführung von Maßnahmen der assistierten Befruchtung ohne ausreichende Nachweise und/oder ohne Teilnahme an den von der Ärztekammer eingeführten Maßnahmen zur Qualitätssicherung ist berufswidrig.

§ 14

Erhaltung des ungeborenen Lebens und Schwangerschaftsabbruch

(1) Ärztinnen und Ärzte sind grundsätzlich verpflichtet, das ungeborene Leben zu erhalten. Der Schwangerschaftsabbruch unterliegt den gesetzlichen Bestimmungen. Ärztinnen und Ärzte können nicht gezwungen werden, einen Schwangerschaftsabbruch vorzunehmen oder ihn zu unterlassen.

(2) Ärztinnen und Ärzte, die einen Schwangerschaftsabbruch durchführen oder eine Fehlgeburt betreuen, haben dafür Sorge zu tragen, dass die tote Leibesfrucht keiner missbräuchlichen Verwendung zugeführt wird.

§ 15

Forschung

(1) Ärztinnen und Ärzte, die sich an einem Forschungsvorhaben beteiligen, bei dem in die psychische oder körperliche Integrität eines Menschen eingegriffen oder Körpermaterialien oder Daten verwendet werden, die sich einem bestimmten Menschen zuordnen lassen, müssen sicherstellen, dass vor der Durchführung des Forschungsvorhabens eine Beratung erfolgt, die auf die mit ihm verbundenen berufsethischen und berufsrechtlichen Fragen zielt und die von einer bei der zuständigen Ärztekammer gebildeten Ethikkommission oder von einer anderen, nach Landesrecht gebildeten unabhängigen und interdisziplinär besetzten Ethikkommission durchgeführt wird. Dasselbe gilt vor der Durchführung gesetzlich zugelassener Forschung mit vitalen menschlichen Gameten und lebendem embryonalen Gewebe.

(2) Ist bereits eine Beratung von ärztlichen Kolleginnen oder Kollegen gemäß Absatz 1 erfolgt, zeigen Ärztinnen und Ärzte ihre Beteiligung an dem Forschungsvorhaben unter Nachweis der erfolgten Beratung bei der für sie nach Landesrecht zuständigen Ethikkommission an.

(3) In Publikationen von Forschungsergebnissen sind die Beziehungen der Ärztin oder des Arztes zum Auftraggeber und dessen Interessen offenzulegen.

(4) Ärztinnen und Ärzte beachten bei der Forschung am Menschen nach § 15 Absatz 1 die in der Deklaration von Helsinki des Weltärztebundes in der Fassung der 75. Generalversammlung 2024 in Helsinki niedergelegten ethischen Grundsätze für die medizinische Forschung am Menschen.

§ 16

Beistand für Sterbende

Ärztinnen und Ärzte haben Sterbenden unter Wahrung ihrer Würde und unter Achtung ihres Willens beizustehen. Es ist ihnen verboten, Patientinnen und Patienten auf deren Verlangen zu töten. Sie dürfen keine Hilfe zur Selbsttötung leisten.[1]

IV. Berufliches Verhalten

1. Berufsausübung

§ 17

Niederlassung und Ausübung der Praxis

(1) Die Ausübung ambulanter ärztlicher Tätigkeit außerhalb von Krankenhäusern einschließlich konzessionierter Privatkliniken ist an die Niederlassung in einer Praxis (Praxissitz) gebunden, soweit nicht gesetzliche Vorschriften etwas anderes zulassen.

(2) Die Ausübung ambulanter ärztlicher Tätigkeit im Umherziehen, in gewerblicher Form oder bei Beschäftigungsträgern, die gewerbsmäßig ambulante heilkundliche Leistungen erbringen, ist berufswidrig, soweit nicht die Tätigkeit in Krankenhäusern oder konzessionierten Privatkrankenanstalten ausgeübt wird oder gesetzliche Vorschriften etwas anderes zulassen.

(3) Auf Antrag kann die Ärztekammer von den Geboten oder Verboten der Absätze 1 und 2 Ausnahmen gestatten, dies gilt auch zum Zwecke der aufsuchenden medizinischen Gesundheitsversorgung, wenn sichergestellt ist, dass die beruflichen Belange nicht beeinträchtigt werden und die Berufsordnung beachtet wird.

(4) Ärztinnen und Ärzten ist es gestattet, über den Praxissitz hinaus an zwei weiteren Orten ärztlich tätig zu sein. Sie haben Vorkehrungen für eine ordnungsgemäße Versorgung ihrer Patientinnen und Patienten an jedem Ort ihrer Tätigkeiten zu treffen.

(5) Der Praxissitz ist durch ein Praxisschild kenntlich zu machen. Ärztinnen und Ärzte haben auf ihrem Praxisschild

- den Namen
- die (Fach-)Arztbezeichnung
- die Sprechzeiten sowie
- ggf. die Zugehörigkeit zu einer Berufsausübungsgemeinschaft gem. § 18 a anzugeben.

---

[1] § 16 Satz 3 in der Fassung vom 16. November 2019 wird durch Beschluss der Kammerversammlung der Ärztekammer Nordrhein vom 13. November 2021 für nicht anwendbar erklärt.

Ärztinnen und Ärzte, welche nicht unmittelbar patientenbezogen tätig werden, können von der Ankündigung ihres Praxissitzes durch ein Praxisschild absehen, wenn sie dies der Ärztekammer anzeigen.

(6) Ort und Zeitpunkt der Aufnahme der Tätigkeiten am Praxissitz sowie die Aufnahme weiterer Tätigkeiten und jede Veränderung haben Ärztinnen und Ärzte der Ärztekammer unverzüglich mitzuteilen.

§ 18

Berufliche Kooperation

(1) Ärztinnen und Ärzte dürfen sich zu Berufsausübungsgemeinschaften, Organisationsgemeinschaften, Kooperationsgemeinschaften und Praxisverbünden zusammenschließen. Der Zusammenschluss zur gemeinsamen Ausübung des Arztberufs kann zum Erbringen einzelner Leistungen erfolgen, sofern er nicht einer Umgehung des § 31 dient. Eine Umgehung liegt insbesondere vor, wenn der Gewinn ohne Grund in einer Weise verteilt wird, die nicht dem Anteil der persönlich erbrachten Leistungen entspricht. Die Anordnung einer Leistung, insbesondere aus den Bereichen der Labormedizin, der Pathologie und der bildgebenden Verfahren, stellt keinen Leistungsanteil im Sinne des Satzes 3 dar. Verträge über die Gründung von Teil-Berufsausübungsgemeinschaften sind der Ärztekammer vorzulegen.

(2) Sie dürfen ihren Beruf alleine oder in Gemeinschaft in allen für den Arztberuf zulässigen Gesellschaftsformen ausüben, wenn ihre eigenverantwortliche, medizinisch unabhängige sowie nicht gewerbliche Berufausübung gewährleistet ist. Bei beruflicher Zusammenarbeit, gleich in welcher Form, hat jede Ärztin und jeder Arzt zu gewährleisten, dass die ärztlichen Berufspflichten eingehalten werden.

(3) Die Zugehörigkeit zu bis zu zwei weiteren Berufsausübungsgemeinschaften im Rahmen des § 17 Abs. 4 ist zulässig. Die Berufsausübungsgemeinschaft erfordert einen gemeinsamen Praxissitz. Eine Berufsausübungsgemeinschaft mit mehreren Praxissitzen ist zulässig, wenn an dem jeweiligen Praxissitz verantwortlich mindestens ein Mitglied der Berufsausübungsgemeinschaft eine ausreichende Patientenversorgung sicherstellt.

(4) Bei allen Formen der ärztlichen Kooperation muss die freie Arztwahl gewährleistet bleiben.

(5) Soweit Vorschriften dieser Berufsordnung Regelungen des Partnerschaftsgesellschaftsgesetzes (Gesetz über Partnerschaftsgesellschaften Angehöriger Freier Berufe [PartGG] vom 25.07.1994 - BGBl. I S. 1744) einschränken, sind sie vorrangig aufgrund von § 1 Abs. 3 PartGG.

(6) Alle Zusammenschlüsse nach Absatz 1 sowie deren Änderung und Beendigung sind der zuständigen Ärztekammer anzuzeigen. Sind für die beteiligten Ärztinnen und Ärzte mehrere Ärztekammern zuständig, so ist jede Ärztin und jeder Arzt verpflichtet, die für ihn zuständige Kammer auf alle am Zusammenschluss beteiligten Ärztinnen und Ärzte hinzuweisen.

§ 18 a

Ankündigung von Berufsausübungsgemeinschaften und sonstigen Kooperationen

(1) Bei Berufsausübungsgemeinschaften von Ärztinnen und Ärzten sind - unbeschadet des Namens einer Partnerschaftsgesellschaft oder einer juristischen Person des Privatrechts - die Namen und Arztbezeichnungen aller in der Gemeinschaft zusammengeschlossenen Ärztinnen und Ärzte sowie die Rechtsform anzukündigen. Bei mehreren Praxissitzen gemäß § 17 Abs. 4 ist jeder Praxissitz gesondert anzukündigen. § 19 Abs. 3 gilt entsprechend. Die Fortführung des Namens einer/eines nicht mehr berufstätigen, ausgeschiedenen oder verstorbenen Partnerin/Partners ist unzulässig.

(2) Bei Kooperationen gemäß § 23 a muss sich die Ärztin oder der Arzt in ein gemeinsames Praxisschild mit den Kooperationspartnern aufnehmen lassen. Bei Partnerschaften gemäß § 23 b darf die Ärztin oder der Arzt, wenn die Angabe seiner Berufsbezeichnung vorgesehen ist, nur gestatten, dass die Bezeichnung "Ärztin" oder "Arzt" oder eine andere führbare Bezeichnung angegeben wird.

(3) Zusammenschlüsse zu Organisationsgemeinschaften dürfen angekündigt werden. Die Zugehörigkeit zu einem Praxisverbund gemäß § 23 c kann durch Hinzufügen des Namens des Verbundes angekündigt werden.

§ 19

Beschäftigung angestellter Praxisärztinnen und -ärzte

(1) Ärztinnen und Ärzte müssen die Praxis persönlich ausüben. Die Beschäftigung ärztlicher Mitarbeiterinnen und Mitarbeiter in der Praxis setzt die Leitung der Praxis durch die niedergelassene Ärztin oder den niedergelassenen Arzt voraus. Die Ärztin oder der Arzt hat die Beschäftigung der ärztlichen Mitarbeiterin oder des Mitarbeiters der Ärztekammer anzuzeigen.

(2) Ärztinnen und Ärzte dürfen nur zu angemessenen Bedingungen beschäftigt werden. Angemessen sind insbesondere Bedingungen, die der beschäftigten Ärztin oder dem beschäftigten Arzt eine angemessene Vergütung gewähren sowie angemessene Zeit zur Fortbildung einräumen und bei der Vereinbarung von Wettbewerbsverboten eine angemessene Ausgleichszahlung vorsehen.

(3) Über die in der Praxis tätigen angestellten Ärztinnen und Ärzte müssen die Patientinnen und Patienten in geeigneter Weise informiert werden.

§ 20

Vertretung

(1) Niedergelassene Ärztinnen und Ärzte sollen grundsätzlich zur gegenseitigen Vertretung bereit sein. Sie dürfen sich grundsätzlich nur durch eine Fachärztin oder einen Facharzt desselben Fachgebiets vertreten lassen. Übernommene Patientinnen und Patienten sind nach Beendigung der Vertretung zurück zu überweisen.

(2) Die Praxis einer verstorbenen Ärztin oder eines verstorbenen Arztes kann zugunsten ihres Witwers oder seiner Witwe, ihrer Partnerin oder seines Partners nach dem Gesetz über die eingetragene Lebenspartnerschaft oder eines unterhaltsberechtigten Angehörigen in der Regel bis zur Dauer von sechs Monaten nach dem Ende des Kalendervierteljahres, in dem der Tod eingetreten ist, durch eine andere Ärztin oder einen anderen Arzt fortgesetzt werden.

§ 21

Haftpflichtversicherung

Ärztinnen und Ärzte sind verpflichtet, sich hinreichend gegen Haftpflichtansprüche im Rahmen ihrer beruflichen Tätigkeit zu versichern.

§ 22

(unbesetzt)

§ 22 a

(unbesetzt)

§ 23

Ärztinnen und Ärzte im Beschäftigungsverhältnis

(1) Die Regeln dieser Berufsordnung gelten auch für Ärztinnen und Ärzte, welche ihre ärztliche Tätigkeit im Rahmen eines privatrechtlichen Arbeitsverhältnisses oder öffentlich-rechtlichen Dienstverhältnisses ausüben.

(2) Auch in einem Arbeits- oder Dienstverhältnis dürfen Ärztinnen und Ärzte eine Vergütung für ihre ärztliche Tätigkeit nicht dahingehend vereinbaren, dass die Vergütung sie in der Unabhängigkeit ihrer medizinischen Entscheidungen beeinträchtigt.

§ 23 a

Kooperationsgemeinschaft zwischen Ärzten und Angehörigen anderer Fachberufe im Gesundheitswesen

(1) Ärztinnen und Ärzte können sich auch mit selbständig tätigen und zur eigenverantwortlichen Berufsausübung befugten Berufsangehörigen anderer akademischer Heilberufe im Gesundheitswesen oder staatlicher Ausbildungsberufe im Gesundheitswesen sowie anderen Naturwissenschaftlerinnen und Naturwissenschaftlern und Angehörigen sozialpädagogischer Berufe - auch beschränkt auf einzelne Leistungen - zur kooperativen Berufsausübung zusammenschließen (medizinische Kooperationsgemeinschaft). Die Kooperation ist in der Form einer Partnerschaftsgesellschaft nach dem PartGG oder aufgrund eines schriftlichen Vertrages über die Bildung einer Kooperationsgemeinschaft in der Rechtsform einer Gesellschaft bürgerlichen Rechts gestattet. Ärztinnen und Ärzten ist ein solcher Zusammenschluss im Einzelnen nur mit solchen anderen Berufsangehörigen und in der Weise erlaubt, dass diese in ihrer Verbindung mit der Ärztin oder dem Arzt einen gleichgerichteten oder integrierenden diagnostischen oder therapeutischen Zweck bei der Heilbehandlung, auch auf dem Gebiete der Prävention und Rehabilitation, durch räumlich nahes und koordiniertes Zusammenwirken aller beteiligten Berufsangehörigen erfüllen können. Darüber hinaus muss der Kooperationsvertrag gewährleisten, dass

a. die eigenverantwortliche und selbständige Berufsausübung der Ärztin oder des Arztes gewahrt ist;

b. die Verantwortungsbereiche der Partner gegenüber den Patientinnen und Patienten getrennt bleiben;

c. medizinische Entscheidungen, insbesondere über Diagnostik und Therapie, ausschließlich die Ärztin oder der Arzt trifft, sofern nicht die Ärztin oder der Arzt nach ihrem oder seinem Berufsrecht den in der Gemeinschaft selbständig tätigen Berufsangehörigen eines anderen Fachberufs solche Entscheidungen überlassen darf;

d. der Grundsatz der freien Arztwahl gewahrt bleibt;

e. die behandelnde Ärztin oder der behandelnde Arzt zur Unterstützung in den diagnostischen Maßnahmen oder zur Therapie auch andere als die in der Gemeinschaft kooperierenden Berufsangehörigen hinzuziehen kann;

f. die Einhaltung der berufsrechtlichen Bestimmungen der Ärztinnen und Ärzte, insbesondere die Pflicht zur Dokumentation, das Verbot der berufswidrigen Werbung und die Regeln zur Erstellung einer Honorarforderung von den übrigen Partnerinnen und Partnern beachtet wird;

g. sich die Kooperationsgemeinschaft verpflichtet, im Rechtsverkehr die Namen aller Partnerinnen und Partner und ihre Berufsbezeichnungen anzugeben und - sofern es sich um eine eingetragene Partnerschaftsgesellschaft handelt - den Zusatz "Partnerschaft" zu führen.

Die Kooperation muss die Namen aller Partnerinnen/Partner einschließlich der ausgeübten Berufe mit dem Zusatz „Kooperationsgemeinschaft“ führen.

(2) Die für die Mitwirkung der Ärztin oder des Arztes zulässige berufliche Zusammensetzung der Kooperation im Einzelnen richtet sich nach dem Gebot des Absatzes 1 Satz 3; es ist erfüllt, wenn Angehörige aus den vorgenannten Berufsgruppen kooperieren, die mit der Ärztin oder dem Arzt entsprechend ihrem oder seinem Fachgebiet einen gemeinschaftlich erreichbaren medizinischen Zweck nach der Art ihrer beruflichen Kompetenz zielbezogen erfüllen können.

§ 23 b

(aufgehoben)

§ 23 c

Praxisverbund

(1) Ärztinnen und Ärzte dürfen, auch ohne sich zu einer Berufsausübungsgemeinschaft zusammenzuschließen, eine Kooperation verabreden (Praxisverbund), welche auf die Erfüllung eines durch gemeinsame oder gleichgerichtete Maßnahmen bestimmten Versorgungsauftrags oder auf eine andere Form der Zusammenarbeit zur Patientenversorgung, z. B. auf dem Felde der Qualitätssicherung oder Versorgungsbereitschaft, gerichtet ist. Die Teilnahme soll allen dazu bereiten Ärztinnen und Ärzten ermöglicht werden; soll die Möglichkeit zur Teilnahme beschränkt werden, z. B. durch räumliche oder qualitative Kriterien, müssen die dafür maßgeblichen Kriterien für den Versorgungsauftrag notwendig und nicht diskriminierend sein und der Ärztekammer gegenüber offengelegt werden. Ärztinnen und Ärzte in einer zulässigen Kooperation dürfen die medizinisch gebotene oder von der Patientin oder dem Patienten gewünschte Überweisung an nicht dem Verbund zugehörige Ärztinnen und Ärzte nicht behindern.

(2) Die Bedingungen der Kooperation nach Absatz 1 müssen in einem schriftlichen Vertrag niedergelegt werden, der der Ärztekammer vorgelegt werden muss.

(3) In eine Kooperation nach Absatz 1 können auch Krankenhäuser, Vorsorge- und Rehakliniken und Angehörige anderer Gesundheitsberufe nach § 23 a einbezogen werden, wenn die Grundsätze nach § 23 a gewahrt sind.

§ 24

Verträge über ärztliche Tätigkeit

Ärztinnen und Ärzte sollen alle Verträge über ihre ärztliche Tätigkeit vor ihrem Abschluss der Ärztekammer vorlegen, damit geprüft werden kann, ob die beruflichen Belange gewahrt sind.

§ 25

Ärztliche Gutachten und Zeugnisse

Bei der Ausstellung ärztlicher Gutachten und Zeugnisse haben Ärztinnen und Ärzte mit der notwendigen Sorgfalt zu verfahren und nach bestem Wissen ihre ärztliche Überzeugung auszusprechen. Gutachten und Zeugnisse, zu deren Ausstellung Ärztinnen und Ärzte verpflichtet sind oder die auszustellen sie übernommen haben, sind innerhalb einer angemessenen Frist abzugeben. Zeugnisse über Mitarbeiterinnen und Mitarbeiter und Ärztinnen und Ärzte in Weiterbildung müssen grundsätzlich innerhalb von drei Monaten nach Antragstellung, bei Ausscheiden unverzüglich, ausgestellt werden.

§ 26

Ärztlicher Notfalldienst

Ärztinnen und Ärzte sind nach Maßgabe des nordrhein-westfälischen Heilberufsgesetzes zur Teilnahme am Notfalldienst verpflichtet.

2. Berufliche Kommunikation

§ 27

Erlaubte Information und berufswidrige Werbung

(1) Zweck der nachstehenden Vorschriften der Berufsordnung ist die Gewährleistung des Patientenschutzes durch sachgerechte und angemessene Information und die Vermeidung einer dem Selbstverständnis der Ärztin/des Arztes zuwiderlaufenden Kommerzialisierung des Arztberufes.

(2) Auf dieser Grundlage sind der Ärztin/dem Arzt sachliche, berufsbezogene Informationen gestattet.

(3) Berufswidrige Werbung ist Ärztinnen und Ärzten untersagt. Ärztinnen/Ärzte dürfen eine solche Werbung weder veranlassen noch dulden. Berufswidrig ist insbesondere eine anpreisende, irreführende oder vergleichende Werbung. Eine Werbung für eigene oder fremde gewerbliche Tätigkeiten oder Produkte im Zusammenhang mit der eigenen ärztlichen Tätigkeit ist unzulässig. Werbeverbote aufgrund anderer gesetzlicher Bestimmungen bleiben unberührt.

(4) Die Ärztin/Der Arzt kann

1. nach der Weiterbildungsordnung erworbene Bezeichnungen,
2. nach sonstigen öffentlich-rechtlichen Vorschriften erworbene Qualifikationen,
3. bis zu drei als solche gekennzeichnete Tätigkeitsschwerpunkte,
4. organisatorische Hinweise

ankündigen.

(5) Die nach Abs. 4 Nr. 1 erworbenen Bezeichnungen dürfen nur in der nach der Weiterbildungsordnung zulässigen Form geführt werden. Ein Hinweis auf die verleihende Ärztekammer ist zulässig. Andere Qualifikationen und Tätigkeitsschwerpunkte dürfen nur angekündigt werden, wenn diese Angaben nicht mit solchen nach geregeltem Weiterbildungsrecht erworbenen Qualifikationen verwechselt werden können. Die Angaben nach Abs. 4 Nrn. 1 und 2 sind nur zulässig, wenn die Ärztin/der Arzt die umfassten Tätigkeiten nicht nur gelegentlich ausübt.

(6) Tätigkeitsschwerpunkte können angekündigt und müssen mit dem Zusatz „Tätigkeitsschwerpunkte“ gekennzeichnet werden. Zur Ankündigung dieser Angaben ist berechtigt, wer diese Leistungen seit mindestens zwei Jahren in erheblichem Umfang erbringt und diese auf Verlangen der Ärztekammer nachweisen kann.

(7) Die Ärztin/Der Arzt hat der Ärztekammer auf deren Verlangen die zur Prüfung der Voraussetzungen der Ankündigung erforderlichen Unterlagen vorzulegen. Die Ärztekammer ist befugt, ergänzende Auskünfte zu verlangen.

§ 28

(aufgehoben)

3. Berufliche ärztliche Zusammenarbeit

§ 29

Kollegiale Zusammenarbeit

(1) Ärztinnen und Ärzte haben sich untereinander kollegial zu verhalten. Die Verpflichtung, in einem Gutachten, auch soweit es die Behandlungsweise von Kolleginnen und Kollegen betrifft, nach bestem Wissen die ärztliche Überzeugung auszusprechen, bleibt unberührt. Unsachliche Kritik an der Behandlungsweise oder dem beruflichen Wissen einer Ärztin oder eines Arztes sowie herabsetzende Äußerungen sind berufswidrig.

(2) Es ist berufswidrig, eine Kollegin oder einen Kollegen aus der Behandlungstätigkeit oder aus dem Wettbewerb um eine berufliche Tätigkeit durch unlautere Handlungen zu verdrängen. Es ist insbesondere berufswidrig, wenn Ärztinnen und Ärzte innerhalb eines Zeitraumes von einem Jahr ohne Zustimmung der Praxisinhaberin oder des Praxisinhabers im Einzugsbereich derjenigen Praxis als Fachärztinnen/Fachärzte ärztlich tätig werden, in welcher sie in der Aus- oder Weiterbildung mindestens 3 Monate tätig waren. Hiervon unberührt bleibt eine Anstellung als Weiterbildungsassistent/in in einem Gebiet zu einer weiteren Facharztqualifikation, einer Schwerpunktkompetenz oder Zusatzweiterbildung. Ebenso ist es berufswidrig, in unlauterer Weise eine Kollegin oder einen Kollegen ohne angemessene Vergütung oder unentgeltlich zu beschäftigen oder eine solche Beschäftigung zu bewirken oder zu dulden.

(3) Ärztinnen und Ärzte mit aus einem Liquidationsrecht resultierenden oder anderweitigen Einkünften aus ärztlicher Tätigkeit (z. B. Beteiligungsvergütung) sind verpflichtet, den von ihnen dazu herangezogenen Kolleginnen und Kollegen eine angemessene Vergütung zu gewähren bzw. sich dafür einzusetzen, dass die Mitarbeit angemessen vergütet wird.

(4) In Gegenwart von Patientinnen und Patienten oder anderen Personen sind Beanstandungen der ärztlichen Tätigkeit und zurechtweisende Belehrungen zu unterlassen. Das gilt auch im Verhältnis von Vorgesetzten und Mitarbeitern und für den Dienst in Krankenhäusern.

(5) Die zur Weiterbildung befugten Ärztinnen und Ärzte haben ihre nach der Weiterbildungsordnung gegenüber Weiterzubildenden bestehenden Pflichten zu erfüllen.

(6) Ärztinnen und Ärzte dürfen ihre Mitarbeiterinnen und Mitarbeiter nicht diskriminieren und haben insbesondere die Bestimmungen des Arbeits- und Berufsbildungsrechts zu beachten.

§ 29 a

Zusammenarbeit mit Dritten

(1) Ärztinnen und Ärzten ist es nicht gestattet, zusammen mit Personen, die weder Ärztinnen oder Ärzte sind, noch zu ihren berufsmäßig tätigen Mitarbeiterinnen oder Mitarbeitern gehören, Patientinnen und Patienten zu untersuchen oder zu behandeln. Dies gilt nicht für Personen, welche sich in der Ausbildung zum ärztlichen Beruf oder zu einem Fachberuf im Gesundheitswesen befinden.

(2) Die Zusammenarbeit mit Angehörigen anderer Fachberufe im Gesundheitswesen ist zulässig, wenn die Verantwortungsbereiche der Ärztin oder des Arztes und des Angehörigen des Fachberufes klar erkennbar voneinander getrennt bleiben.

4. Wahrung der ärztlichen Unabhängigkeit bei der Zusammenarbeit mit Dritten

§ 30

Ärztliche Unabhängigkeit

Ärztinnen und Ärzte sind verpflichtet, in allen vertraglichen und sonstigen beruflichen Beziehungen zu Dritten ihre ärztliche Unabhängigkeit für die Behandlung der Patientinnen und Patienten zu wahren.

§ 31

Unerlaubte Zuweisung

(1) Ärztinnen und Ärzten ist es nicht gestattet, für die Zuweisung von Patientinnen und Patienten oder Untersuchungsmaterial oder für die Verordnung oder den Bezug von Arznei- oder Hilfsmitteln oder Medizinprodukten ein Entgelt oder andere Vorteile zu fordern, sich oder Dritten versprechen oder gewähren zu lassen oder selbst zu versprechen oder zu gewähren.

(2) Sie dürfen ihren Patientinnen und Patienten nicht ohne hinreichenden Grund bestimmte Ärztinnen oder Ärzte, Apotheken, Personen oder Unternehmen, die Heil- und Hilfsmittel erbringen oder sonstige gesundheitliche Leistungen anbieten, empfehlen oder an diese verweisen.

§ 32

Unerlaubte Zuwendungen

(1) Ärztinnen und Ärzten ist es nicht gestattet, von Patientinnen und Patienten oder Anderen Geschenke oder andere Vorteile für sich oder Dritte zu fordern oder sich oder Dritten versprechen zu lassen oder anzunehmen, wenn hierdurch der Eindruck erweckt wird, dass die Unabhängigkeit der ärztlichen Entscheidung beeinflusst wird. Eine Beeinflussung ist dann nicht berufswidrig, wenn sie einer wirtschaftlichen Behandlungs- oder Verordnungsweise auf sozialrechtlicher Grundlage dient und der Ärztin oder dem Arzt die Möglichkeit erhalten bleibt, aus medizinischen Gründen eine andere als die mit finanziellen Anreizen verbundene Entscheidung zu treffen.

(2) Die Annahme von geldwerten Vorteilen in angemessener Höhe ist nicht berufswidrig, sofern diese ausschließlich für berufsbezogene Fortbildung verwendet werden. Der für die Teilnahme an einer wissenschaftlichen Fortbildungsveranstaltung gewährte Vorteil ist unangemessen, wenn er über die notwendigen Reisekosten und Tagungsgebühren hinausgeht.

(3) Die Annahme von Beiträgen Dritter zur Durchführung von Veranstaltungen (Sponsoring) ist ausschließlich für die Finanzierung des wissenschaftlichen Programms ärztlicher Fortbildungsveranstaltungen und nur in angemessenem Umfang erlaubt. Das Sponsoring ist bei der Ankündigung und Durchführung der Veranstaltung offenzulegen.

§ 33

Zuwendungen bei vertraglicher Zusammenarbeit

Soweit Ärztinnen und Ärzte Leistungen für Dritte im Bereich Arznei- oder Hilfsmittel, Medizinprodukte oder in der Heilmittelversorgung erbringen (z. B. bei Anwendungsbeobachtungen), muss die hierfür bestimmte Vergütung der erbrachten Leistung entsprechen. Die Annahme von Werbeabgaben oder von Vorteilen für den Besuch von Informationsveranstaltungen Dritter im Sinne von Satz 1 ist untersagt, sofern der Wert nicht geringfügig ist.

§ 34

(aufgehoben)

§ 35

(aufgehoben)

C. Verhaltensregeln (Grundsätze korrekter ärztlicher Berufsausübung)

Nr. 1
(aufgehoben)

Nr. 2
(aufgehoben)

Nr. 3
(aufgehoben)

D. Ergänzende Bestimmungen zu einzelnen ärztlichen Berufspflichten

I. Pflichten bei grenzüberschreitender ärztlicher Tätigkeit

Nr. 1
(aufgehoben)

Nr. 2
(aufgehoben)

II. Pflichten in besonderen medizinischen Situationen

Nr. 3
(aufgehoben)

Nr. 4
(aufgehoben)

E. Anlage

R i c h t l i n i e
zur Durchführung der assistierten Reproduktion
gemäß § 13 Berufsordnung für die nordrheinischen Ärztinnen und Ärzte

(aufgehoben)
