# Supplementary material for: Procalcitonin as a predictor of length of stay in patients with odontogenic infections
Source: Oral Maxillofac Surg. 2026 Aug 7;30(1):128. doi: 10.1007/s10006-026-01617-6 (PMC13451498; doi:10.1007/s10006-026-01617-6)
Supplement: Supplementary file 3 [file 10006_2026_1617_MOESM3_ESM.pdf]

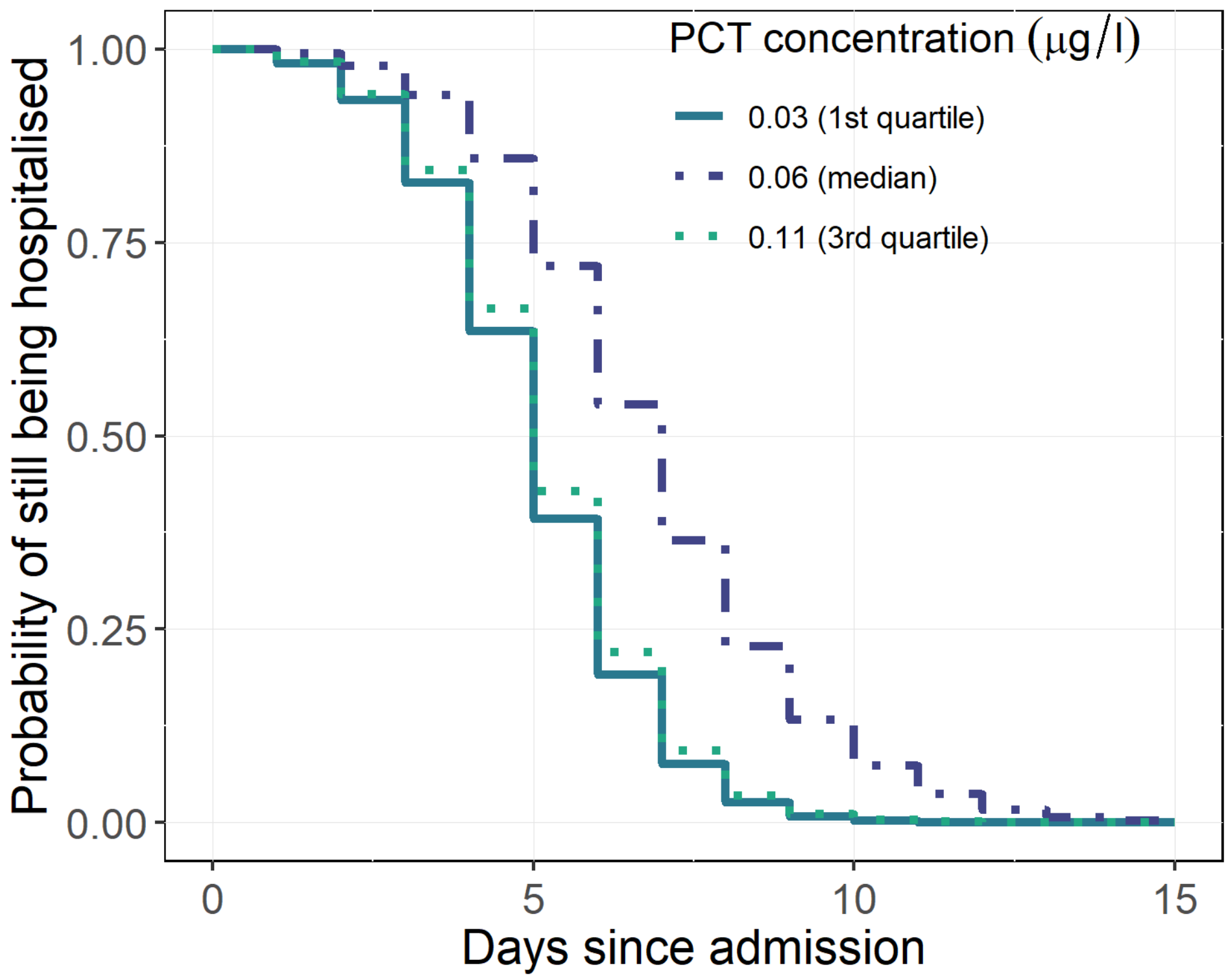

PCT concentration (μg/l)
0.03 (1st quartile)
0.06 (median)
0.11 (3rd quartile)
Probability of still being hospitalised
1.00
0.75
0.50
0.25
0.00
0
5
10
15
Days since admission
